# Patient-Specific Bone Multiscale Modelling, Fracture Simulation and Risk Analysis—A Survey

**DOI:** 10.3390/ma13010106

**Published:** 2019-12-24

**Authors:** Amadeus C. S. Alcântara, Israel Assis, Daniel Prada, Konrad Mehle, Stefan Schwan, Lúcia Costa-Paiva, Munir S. Skaf, Luiz C. Wrobel, Paulo Sollero

**Affiliations:** 1 Department of Computational Mechanics, School of Mechanical Engineering, University of Campinas—UNICAMP, Campinas, Sao Paulo 13083-860, Brazil; amadeus.alcantara@fem.unicamp.br (A.C.S.A.); daniel.prada@fem.unicamp.br (D.P.); 2 Department of Integrated Systems, School of Mechanical Engineering, University of Campinas—UNICAMP, Campinas, Sao Paulo 13083-860, Brazil; israel.dynamics@gmail.com; 3 Department of Engineering and Natural Sciences, University of Applied Sciences Merseburg, 06217 Merseburg, Germany; konrad.mehle@hs-merseburg.de; 4 Fraunhofer Institute for Microstructure of Materials and Systems IMWS, 06120 Halle/Saale, Germany; stefan.schwan@imws.fraunhofer.de; 5 Department of Obstetrics and Gynecology, School of Medical Sciences, University of Campinas—UNICAMP, Campinas, Sao Paulo 13083-887, Brazil; paivaepaiva@uol.com.br; 6 Institute of Chemistry and Center for Computing in Engineering and Sciences, University of Campinas—UNICAMP, Campinas, Sao Paulo 13083-860, Brazil; skaf@unicamp.br; 7 Institute of Materials and Manufacturing, Brunel University London, Uxbridge UB8 3PH, UK; Luiz.Wrobel@brunel.ac.uk; 8 Department of Civil and Environmental Engineering, Pontifical Catholic University of Rio de Janeiro, Rio de Janeiro 22451-900, Brazil

**Keywords:** bone fracture, patient-specific bone models, osteoporosis, bone multiscale structure, bone multiscale modelling, fracture risk analysis

## Abstract

This paper provides a starting point for researchers and practitioners from biology, medicine, physics and engineering who can benefit from an up-to-date literature survey on patient-specific bone fracture modelling, simulation and risk analysis. This survey hints at a framework for devising realistic patient-specific bone fracture simulations. This paper has 18 sections: Section 1 presents the main interested parties; Section 2 explains the organzation of the text; Section 3 motivates further work on patient-specific bone fracture simulation; Section 4 motivates this survey; Section 5 concerns the collection of bibliographical references; Section 6 motivates the physico-mathematical approach to bone fracture; Section 7 presents the modelling of bone as a continuum; Section 8 categorizes the surveyed literature into a continuum mechanics framework; Section 9 concerns the computational modelling of bone geometry; Section 10 concerns the estimation of bone mechanical properties; Section 11 concerns the selection of boundary conditions representative of bone trauma; Section 12 concerns bone fracture simulation; Section 13 presents the multiscale structure of bone; Section 14 concerns the multiscale mathematical modelling of bone; Section 15 concerns the experimental validation of bone fracture simulations; Section 16 concerns bone fracture risk assessment. Lastly, glossaries for symbols, acronyms, and physico-mathematical terms are provided.

## 1. Interested Parties Concerning this Survey

This paper surveys a multidisciplinary topic. Bone is a not fully understood biological material. Professionals from biology and medicine have been working with physicists and engineers to better understand bone mechanical properties with the goal of anticipating and preventing bone fracture. Bone diseases, specially osteoporosis, have proven to be a major health concern. Governments and philanthropists are putting more effort into minimizing the social and financial consequences of bone fragility fractures. Most fragility fractures are impact-induced fractures occurring in the elderly when performing routine activities. Table 1 shows the main interested parties involved in bone fracture risk analysis and which sections of this paper are most interesting for each party.

## 2. Reading this Paper—Textual Organization and Notation

Throughout this paper, six extra text environments are used to organize ideas intuitively and to facilitate the reading.


**Characteristic Length**
Numerical values, surveyed from the literature, that characterize major geometric features of a specific bone lengthscale, see Definition 8.
**Definition**
Non-mathematical definitions that may be differently understood by specialists from different fields. Mathematical definitions are not presented here due to their complexity. Rigorous mathematical definitions are found in the references present in Appendix C.
**Highlight**
A statement that plays a major role in the argumentation.
**Illustration**
A non-mathematical explanation of a physical phenomenon.
**Open Issue**
Issues and problems not clearly defined or completely solved within the surveyed literature.
**Remark**
Relevant notes.

To make the text less wordy and to shorten sentences whenever possible, a notation has been devised. For instance: $SA_{i}$ represents the i-th concept within the concept group A in section S.

**Figure 1:**
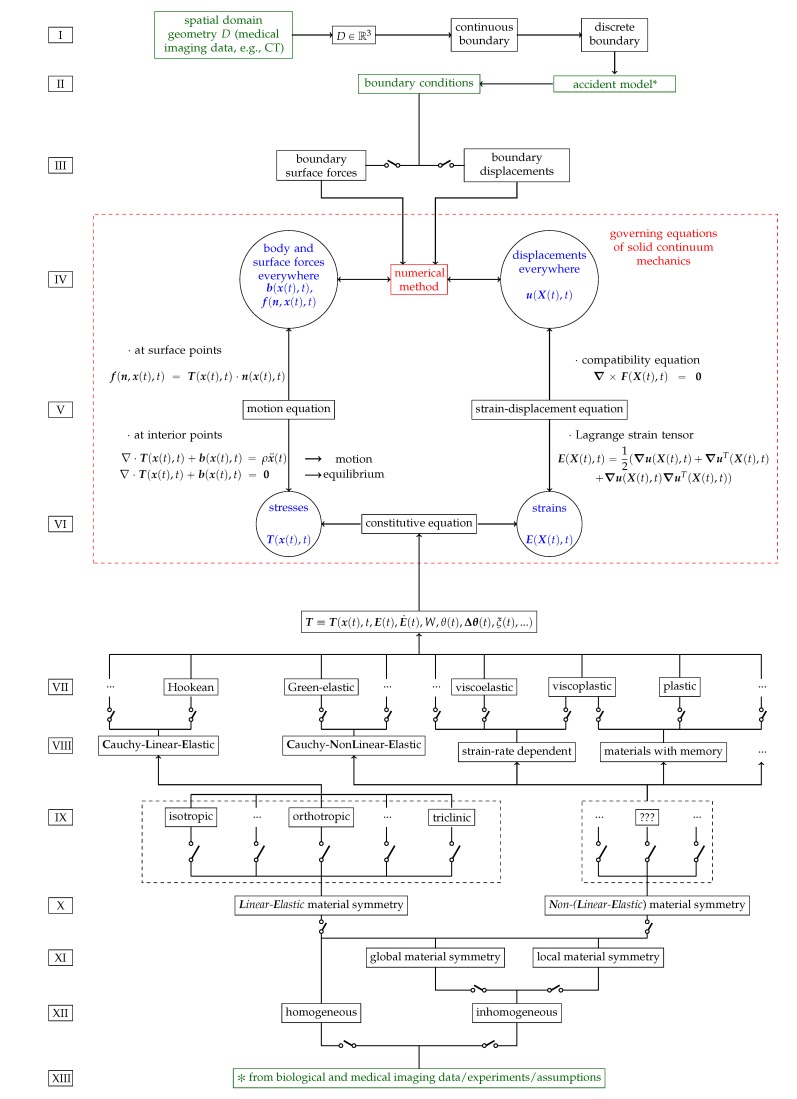
Modelling of a solid continuum mechanics problem for bone.

Some figures and tables are gridded in rows and columns, which, in turn, are labeled by either numbers or letters. For instance, the label Figure 1 row XII refers to the solid’s homogeneity (a solid continuum can be either homogeneous or inhomogeneous); Table 2(1A) refers to the box at column 1 and row A in Table 2, which is titled “**CT**”.

At the end of this paper, glossaries for symbols, acronyms, and physico-mathematical terms are provided, see Appendix A, Appendix B and Appendix C.

## 3. Motivating Patient-Specific Bone Fracture Simulation

An increase in life expectancy implies that the elderly gradually compose a larger fraction of the population. Thus, diseases common among the elderly, e.g., osteoporosis, will occur more frequently, leading to an increase in occurrence of bone fragility fracture. The following Section 3.1, Section 3.2 and Section 3.3 present evidence for this argument.

### 3.1. Ageing Population

Life expectancy has been increasing by about 3 years per decade since 1950 [1]. Due to accelerated scientific and technological development, countless medical breakthroughs have been achieved, enabling people worldwide to live longer [2].

Projections show an intensified increase in the elderly population worldwide. While data from 2018 shows that only 13 countries have at least 20% of their population aged 65 or older, 82 countries are projected to have at least 20% of their population in the same age bracket by 2050 [3]. Furthermore, about 16% of the total world population in 2050 will be aged 65 and older, compared to 9% in 2018 [3]; about 1.5 billion of a projected population of 9.5 billion will be 65 and older in 2050. These projections are confirmed by other reports [4,5].

Analysing individual groups of countries, these same conclusions can be drawn in different magnitudes. A brief compilation of projections on the ageing population of America, Europe and Asia is presented in the next six paragraphs.

*Brazil*—by 2050, the number of people over 50 years old will represent 37% of the population [6]. In parallel, the population over 70 will increase by about 314% in comparison to 2011.

*Latin America*—in the 3 economies with the highest GDP after Brazil, the population over 70 will increase: by 321% in Mexico, by 129.6% in Argentina and by 348% in Colombia [6].

*The United Kingdom*—24% of the population will be 65 years and older in 2037 [7], taking the ≥65 years age bracket to 20.4 million people.

*Europe*—29% of the population will belong to the ≥65 age bracket in 2050, vs. 19% in 2016. In parallel, 13% of the population will be above 80 vs. 4% in 2016 [8].

*The United States of America*—exhibits a trend similar to that of Latin America and Europe [9].

*Asia*—will concentrate 62.3% of the world population in the ≥65 age bracket by 2050 [10].

### 3.2. Osteoporosis—A Major Health Concern

A longer life does not necessarily translate into a better life. The rising average life expectancy implies that a greater fraction of the population will be afflicted by several aging-related bone diseases. In particular, by osteoporosis, a chronic condition characterized by low Bone Mineral Density (BMD) and a consequent increase in fragility fracture risk [11,12]. Menopausal women are major victims of osteoporosis [13,14,15].

Understanding the mechanical behaviour of bone under osteoporosis and conditions such as weightlessness [16,17], radiation and vitamin D deficiency is of vital interest, for instance, to the cause of manned space travel.

**Highlight** **1.** *This paper focuses on osteoporosis, which is the most frequent aging-related bone disease. Other bone diseases not addressed by this paper can be studied by applying the same methodology.*

### 3.3. Osteoporosis—Consequences and Costs

Bone fragility fractures are costly to treat. The associated decrease in quality of life implies further social and financial burden. Besides physical pain and disability, these fractures often lead to clinical death [18,19]. The treatment of bone diseases, e.g., osteoporosis, involves medical imaging, diagnosis and pharmacotherapy [20]. In case of fracture, surgery is often mandatory, implying hospitalization and rehabilitation. These fracture-triggered events produce a chain of financially heavy costs.

It is unsettling that osteoporosis can be clinically silent. As an example, it has been demonstrated that, without prevention and early diagnosis, the costs of osteoporotic fracture-related morbidity and mortality will burden the U.S. health-care system [21]. Logically, if preventive diagnosis methodologies are ignored, the burdening of every health-care systems worldwide is to be expected.

**Highlight** **2.** *If current predictive diagnosis techniques remain unimproved, bone fragility fracture will become a heavier financial burden for health care systems and a social hindrance for people worldwide [22,23,24].*

## 4. Motivating This Literature Survey

Given Highlight 1, it immediately follows the question $4A_{0}$.

$4A_{0}$: How to accurately predict bone fragility fracture?

The definitive answer to $4A_{0}$ is unknown. Trying to answer $4A_{0}$, this paper makes the assumptions $4B_{1}$–$4B_{4}$.

$4B_{1}$: It is possible to use bone fracture simulations to identify bone structure failure criteria.

$4B_{2}$: It is possible to correlate bone structure failure criteria to early signs of bone deterioration.

$4B_{3}$: It is possible to correlate early signs of bone deterioration to bone fracture risk.

$4B_{4}$: It is possible to predict fragility fractures from bone fracture risk analysis.

If $4B_{1}$–$4B_{4}$ are true, improvement in predictive diagnosis is an immediate consequence and part of the unknown answer to $4A_{0}$ lies in answering questions $4A_{1}$–$4A_{3}$.

$4A_{1}$: How to simulate bone fracture?

$4A_{2}$: How to estimate fracture risk?

$4A_{3}$: How to combine the answers to $4A_{1}$–$4A_{2}$ to answer $4A_{0}$?

Any coarse literature review indicates that several approaches have been applied to answer $4A_{1}$–$4A_{2}$. Furthermore, there has not been enough effort to answer $4A_{3}$. Making a single literature survey encompassing the papers that answer $4A_{1}$–$4A_{2}$ is this paper’s contribution to answering $4A_{3}$.

The surveyed literature was critically revisited and herein summarized in a structured view, updating specialists, those listed in Table 1, on bone fracture modelling, simulation and risk analysis. Ultimately, every specialist must: be aware of pertinent open issues and know how to work with other specialists in order to collectively propose solutions.

## 5. Collecting Bibliographical References

This survey was performed using the platforms: *Web of Science*, *Scopus*, *PubMed* and *Google Scholar*. Some of the keywords searched in these platforms were: bone fracture, osteoporosis, bone mechanical properties, bone multiscale modelling, patient-specific bone model, multiscale bone analysis, fracture risk analysis. Papers suggested by these searches as well as other papers referenced by them were analyzed and selected for this survey.

Bone fracture simulation with risk analysis, like any other multidisciplinary topic, see, for instance , ref. [25], exhibits a vast array of relevant papers and applications. Thus, only a limited number of papers could be reviewed. The non-citation of a particular paper is unrelated to the paper’s merit.

**Highlight** **3** (Usage of the Word *Model*). *The word model has different meanings depending on the context. In this paper, a mathematical model is labelled “a model”, i.e., “to model” means “to devise a mathematical model”; a computer-based likeness of a bone sample is referred to as “a geometry model”.*

**Highlight** **4** (Usage of the Word *Simulation*). *In this paper, performing mathematical calculations is labelled “simulation”.*

**Highlight** **5** (The Focus of This Survey). *The focus of this survey lies in papers that applied physico-mathematical approaches to bone fracture modelling and simulation.*

## 6. The Physico-Mathematical Approach to Bone Fracture

Physics and mathematics provide the most accurate descriptions of natural phenomena [26]. Accurate prediction of bone fragility fracture requires such mathematical and physical description. This description consists of equations that have physical, and, thus, biological, interpretations.

Continuum mechanics is a mature field of research and, for this reason, the ubiquitous theory when studying deformation and fracture processes. Continuum mechanics assumes the material to be continuous, i.e., a continuum: between every two points in the spatial domain of the body there always exists another point. A rigorous formulation of a continuum is founded upon, among other mathematical concepts, the basis coordinate frame axes’s Dedekind-density and on Cauchy-continuity [27].

**Remark** **1** (Continuum Mechanics Concepts). *Appendix C presents the list of references for physical and mathematical enunciations to the continuum mechanics concepts used in the text. Biologico-medical concepts can only be incorporated into computer simulations by means of adequate mathematical objects. The mathematical objects required by a consistent physico-mathematical description of bone fracture must mimic physical reality as much as possible.*

## 7. Modelling Bone as a *Continuum*

Figure 1 schematizes the possibilities for modelling patient-specific bone as a continuum and for evaluating, over the entire bone sample, the stresses and strains caused by imposed boundary conditions. Thus, Figure 1 presents what is required to model and simulate patient-specific bone fracture.

Figure 1, inspired by [28] (p. 145), is divided into 13 rows, from I to XIII.

- Row I of Figure 1 illustrates the modelling of patient-specific bone geometry and is further described in Section 9. The spatial domain plus the boundary of a solid bone constitutes its geometry.

**Remark** **2** (Continuous Boundary). *In this paper, the term continuous boundary is used to describe the finest possible geometry discretization given by the resolution of the medical imaging to a computer geometry model. In fact, all geometry models created from medical imaging techniques have a discrete resolution, i.e., a  finite number of pixels/voxels.*

Domain and boundary are mathematically defined in the references of Appendix C, but may not be clear for specialists who are not used to definitions of Partial Differential Equations (PDEs) and continuum mechanics.

**Illustration** **1** (Domain and Boundary). *Picture a potato. In continuum mechanics, the spatial domain of the body represents the interior of the potato. The boundary of the body represents the potato’s skin. Domain plus boundary comprise the entire body.*

- Row II of Figure 1 illustrates the derivation of boundary conditions from accident models which are further described in Section 11.

It is at the boundary of the body, through boundary conditions, where bone models can use information provided by accident models representative of common physical trauma among the elderly, see Section 11. The definition of boundary condition in the context of PDEs may not be clear for non-specialists. In the bone fracture literature, boundary conditions are often labelled as loading or constraint conditions.

**Illustration** **2** (Boundary Condition). *Boundary conditions (BCs) represent the effects of the exterior domain (everything that is not the body) onto the body. BCs describe the interactions between the domain of the body and the exterior domain through the boundary. In structural analysis, BCs are usually represented by surface forces (or tractions) and displacements [29] (p. 11). Boundary conditions require a predefined domain, i.e., geometry. In the diagram of Figure 1 BCs transmit information about the geometry to the set of governing equations, see Section 12, between rows IV and VI.*

- Row III of Figure 1 illustrates the two types of BCs for solid continuum mechanics problems which are briefly described in Section 11.
- Row IV of Figure 1 illustrates the inputs (body forces in the domain, if present, and surface forces and displacements on the boundary, i.e., as BCs) and outputs (surface forces and displacements everywhere) of solid continuum mechanics problems which are further discussed in Section 11.
- Row V of Figure 1 illustrates the motion and strain-displacement equations which are further described in Section 12.
- Row VI of Figure 1 illustrates the relationships between stresses and strains given by constitutive equations which are further described in Section 10.
- Rows VII to VIII of Figure 1 illustrate different types and categorizations of constitutive equations which are further described in Section 10.

**Remark** **3.** (On the Reticences Displayed in the Scheme of Figure 1).*Theorists and experimentalists in the field of mechanics of materials should consider any possible material symmetry and constitutive equation, see $15A_{1}$. The most well-known constitutive equations can only describe bone behaviour up to a certain accuracy. Indeed, if a key variable of bone mechanics is not being considered, it is possible that numerical calculations will never match experimental results. Time, position within the material’s spatial domain, strain, strain rate, temperature, temperature gradients and strain-energy are only a few of the variables that may have an influence on the mechanical behaviour of a material, e.g., bone.*

- Rows IX to X of Figure 1 illustrate different types and categorizations of material symmetry regarding mechanical properties which are further described in Section 10.
- Rows XI to XII of Figure 1 illustrate homogeneity regarding mechanical properties, i.e., material symmetry and constitutive equations, which are further described in Section 10.
- Row XIII of Figure 1 illustrates the experiment-based categorization of mechanical properties, i.e., homogeneity, material symmetry and constitutive equations, which are further described in Section 10, Section 13 and Section 15.

Green coloured texts illustrate the inputs of the modelling process.

Blue coloured texts illustrate the output of the simulation.

Red coloured texts illustrate where the simulation is performed.

Switches in parallel indicate that only one box at a time must be selected when modelling a solid continuum, e.g., a material is either orthotropic or isotropic, but not simultaneously both. Note that inhomogeneous materials are, in fact, globally inhomogeneous, but locally homogeneous. For each homogeneous subdomain of a globally inhomogeneous material, a material symmetry and a constitutive equation must be selected.

Each stage on the diagram of Figure 1 must be modelled and validated as accurately and realistically as possible, so the final results can be used in medical clinics.

**Remark** **4** (Model Accuracy and Validation). *Geometry, discretization, mechanical properties, fall model, boundary conditions, evaluation of stresses, strains, forces and displacements. Accuracy and validation of the current stage on the diagram of Figure 1 depends directly on the accuracy and validation of the previous stage of the diagram, see Section 15.*

One of the main objectives of the continuum mechanics is to study how solids, subjected to certain BCs (e.g., external forces), deform and move. This is achieved through the coupling of strain-displacement, equilibrium and constitutive equations (the governing equations), see Figure 1 and Section 12. The references in Appendix C give more detailed information on continuum mechanics.

## 8. Categorizing the Surveyed Literature into a Continuum Mechanics Framework

Most surveyed papers performing bone simulations, patient-specific or not, of fracture or not, modelled bone as a continuum, following a strategy similar to the diagram in Figure 1. The surveyed literature was categorized regarding its bone computational modelling. The assumptions and considerations made by each reference are registered in Table 2, where tendencies and possible open issues become visible.

Table 2 categorizes the surveyed literature regarding 8 different aspects of solid continuum mechanics modelling. Each column regards one of these aspects:

- Column 1 of Table 2 categorizes the surveyed literature based on how bone geometry is modelled, see Section 9.
- Column 2 of Table 2 categorizes the surveyed literature based on if the inertial term of the motion equation was neglected or not, see Section 12.
- Column 3 of Table 2 categorizes the surveyed literature based on if the non-linear term of the strain-displacement equation was neglected or not, see Section 12.
- Column 4 of Table 2 categorizes the surveyed literature regarding the constitutive equation of their models, see Section 10.
- Column 5 of Table 2 categorizes the surveyed literature regarding the material symmetry of their models, see Section 10.
- Column 6 of Table 2 categorizes the surveyed literature based on the homogeneity regarding the mechanical properties of their models, see Section 10
- Column 7 of Table 2 categorizes the surveyed literature based on the source of the mechanical properties of their models, see Section 10.
- Column 8 of Table 2 categorizes the surveyed literature based on the BCs imposed on their models, see Section 11.

Some references may be repeated within a column in Table 2.

**Remark** **5.** (Multiplicity of References in Table 2). *Reasons for repeating references in the same column are: (**1.**) There is more than one model in the reference, each set up differently; (**2.**) The reference considers a multiscale model and each lengthscale is modelled differently (**3.**) The model is inhomogeneous and each locally homogeneous subdomain is modelled differently.*

The authors tried their best at categorizing each analyzed reference into the most appropriate row of Table 2. However, some references do not clearly or directly reveal how they created their model. Sometimes a bit of interpretation and common sense of the authors was needed to find the best suitable categorization for each reference.

**Remark** **6** (Lack of Clarity). *It is worth to note that some references do not specify their models, see Table 2 row F. The boxes “Unspecified/Other” indicate that the analyzed reference either does not specify its model creation/assumption or uses another, less usual, procedure/assumption not shown in Table 2. The vast majority of references in these boxes do not specify their models. It is very likely that these references assumed the most mature and easy-to-implement modelling strategy. For example, the material was assumed isotropic when no material symmetry was specified, see Table 2 column 5. Complex models tend to be described in detail. Very uncommon assumptions are highlighted throughout this survey.*

Remark 6 leads to Remark 7.

**Remark** **7** (Unifying Framework for Bone Continuum Modeling). *The large number of unspecified modelling data suggests that the literature in the field of bone fracture simulation should be more clear and direct about their modelling. A brief paragraph, or even better a table, addressing all information for continuum modelling shown in Table 2 should be present. In addiction, further information on used mechanical properties and numerical techniques, as shown in Table 2 of [148], would facilitate the understanding and reproducibility of the simulations.*

In addition to assuming bone as a continuum, several of the reviewed papers considered the multiscale structure of bone, see Section 13, and modelled bone as a multiscale material, see Section 14. At the molecular lengthscale, however, bone is not a continuum, but rather a discrete material, or a non-continuum, constituted of molecules, and atoms connected by chemical bonds. Between atoms there is vacuum. Bone is mostly made of vacuum. However, continuum mechanics does not consider the empty spaces between the atoms and is, thus alone not able to foresee where a gummy-bear will break since a continuum has no weakness [149]. Cracks must be first artificially created before propagated.

New multiscale approaches considering molecular structures have arisen to improve the accuracy and precision of material behaviour’s prediction. In the ideal case, bones are modelled as a bunch of (countless) interacting atoms. Nonetheless, this is computationally very expensive and currently impracticable. Today’s greatest clusters can simulate, in a reasonable time, no more than very small cubes with millions, maybe billions of atoms.

As once said by the mathematician Terence Tao: “*Well, just about any useful mathematical model makes non-physical assumptions—for instance, fluids are almost always modelled by a continuum, when in reality they are composed of a huge number of interacting particles. But if the model is robust enough, one can still expect it to give an accurate prediction of reality, even if at an ontological level it is quite distinct*” (https://terrytao.wordpress.com/2011/08/04/localisation-and-compactness-properties-of-the-navier-stokes-global-regularity-problem/).

Physicists and engineers should try to take meaningful *macroscale* information from reduced *molecular* simulations, rather than to simulate all particles within the bone. Continuum mechanics has proved itself to be, in many applications, e.g., fracture mechanics, “robust enough”.

**Remark** **8** (Challenges & Limits). *Current Limits of the Patient-Specific Fracture Simulation are:*

*$6A_{1}$: viability of in vivo experiments for the assessment of patient-specific bone mechanical properties, see Section 10 and $15A_{1}$;*

*$6A_{2}$: viability of in vivo experiments for validation of computer simulations, see $15A_{2}$;*

*$6A_{3}$: computing processor and memory for simulation (mesh refinement increases the number of DOF and so the computing time. Molecular modelling requires an almost infinite number of DOF).*

## 9. Patient-Specific Geometry of Bone

Every bone is unique in its geometry and mechanical properties. Bone fracture models cannot be generalized for all bones, i.e., fracture risk and traction and displacement fields evaluated within the geometry of a specific bone for a given set of BCs are not interchangeable with other bones.

**Illustration** **3.** *Two equally manufactured steel beams behave (at least quasi) equally when subjected to equal BCs. The mathematical description of bone behavior, however, may depend on an infinite number of variables, since there are infinitely possible constitutive equations, relevant physical properties and environmental, e.g., host tissue conditions. Most of the variables considered in mathematical descriptions of living tissue are time-varying and depend on biochemical complexity, animal and human habits, interaction with the environment. For instance, bone undergoes a continuous remodelling process, see Remark 19.*

Thus, bones are *patient-specific*. Each bone is differently “manufactured”.

**Definition** **1** (Patient-Specific: in vivo vs. in vitro). *The term patient-specific denotes each bone in each patient is unique in its geometry and mechanical properties. Thus, bone geometry and mechanical properties are directly assessed from the studied bone. Patient-specific refers to a bone of a living patient, i.e., to in vivo bone. In vivo indicates bone from inside a living organism. Dual to in vivo experiments, in vitro bone experiments are more feasible. In vitro indicates bone outside a living organism.*

Patient-specific: some models can be it, some models cannot be it.

**Remark** **9** *Patient-specific data comes from non-destructive techniques. For instance, the geometry of computational bone models is acquired through non-destructive and non-intrusive medical imaging techniques [50,150]. When not possible, e.g., due to insufficient resolution for specific lengthscale dimensions, to acquire certain patient-specific information, data usually comes from experiments or physical assumptions. For instance, ref. [133] constructs an inhomogeneous, see section Section 10, simulation domain at the microscale, see Section 13, based on simplifying geometric assumptions derived from microscopy.*

**Definition** **2** (Computational Bone Model). *A computational bone model refers to computer files that contain bone geometry and mechanical properties data.*

**Open Issue** **1.** *The validation of patient-specific bone fracture simulations is a major challenge in the field of biomechanics, see Section 15. In vitro experiments do not validate patient-specific bone. However, since there is still no possible way of validating bone computational models by performing in vivo experiments, in vitro experiments are the best way to compare simulations with the real-world.*

Subject-specific and specimen-specific are not the same as patient-specific.

**Definition** **3** (Subject-Specific and Specimen-Specific). *Differently from patient-specific, both subject-specific and specimen-specific terminologies refer either to in vivo non-human bones or to in vitro-experimented bones. A given bone fracture simulation methodology may be applied to bone samples designated by any of the three aforementioned terminologies.*

This section features three non-invasive medical imaging techniques plus microscopy, which is not patient-specific, but is commonly used to model bone micro- and lower-scales-geometry, see Definition 9:

**Computed Tomography** (CT), or X-ray computed tomography, is the most used medical imaging technique among the surveyed literature, as demonstrated in Table 2. CT is argued to be the most accurate 3D medical imaging technique for the creation of computational bone models [151]. However, CT is not recommended for routine clinical examinations due to associated high radiation dosages [152].

**Remark** **10** (CT Resolution). *Ordinary CT-scans have a limited spatial resolution of about 0.5 mm [153]. Thus, they are unable to delineate bone geometry at the microscale, see Section 13. Microscale geometry of bones can be assessed through higher resolution CT techniques, such as High-Resolution peripheral-CT (HR-pCT) and Micro Computed Tomography (µCT).*

***HR-pCT*** *is a high-radiation CT restricted to the peripheral sites of the body, e.g., distal skeleton. HR-pCT provides in vivo imaging with spatial resolution smaller than 100 µm [75,81,153,154]. Similarities between the micro-geometry of peripheral bones and the micro-geometry of non-peripheral bones were discussed and considered by [154,155]. Though HR-pCT is being increasingly used for in vivo bone research, its use has been limited to the distal radius and tibia [156].*

***µCT*** *features a spatial resolution of about 1 µm, higher than that of HR-pCT, enabling delineation of the trabecular microstructure [157,158]. Due to high associated radiation dosages, its usage is restricted to biopsy specimens [153,159]. In comparison to HR-pCT, µCT captures the trabecular porosity more accurately [160].*

*The reviewed literature features a certain confusion between the usage of HR-pCT and µCT terminologies [161]. For instance, it could be argued that [159,162] misplaces terminology. HR-pCT reaches at most ∼ 10 µm; its associated radiation dosage is small enough to allow in vivo rapid tests and is capable of providing a detailed analysis of bone morphology, i.e., geometry [153,161]. µCT displays a finer resolution; its associated radiation dosage restricts applicability to in vitro analysis [161].*

**Remark** **11** (QCT and HR-pQCT). *The Q in QCT and HR-pQCT stands for Quantitative and indicates that a calibration phantom is included in the scanning for the calculation of BMD. However, if a computational model is not aimed, it is preferable to calculate BMD using DXA, see below, because it is more accessible and less expensive [163].*

**Dual-Energy X-ray Absorptiometry** (DEXA, DXA) is the clinical standard to diagnose osteoporosis and fracture risk by measuring areal Bone Mineral Density (aBMD) [97,164,165]. DXA can also contain non-BMD parameters that are correlated to bone fracture [128,166]. The main advantage of DXA over CT is that DXA requires minimal radiation exposure. However, ref. [97,163,167] present a “3D-DXA” method capable of assessing the bone femoral shape and density distribution from 2D DXA images. They are based on statistical shape and appearance models and show good correlations between 3D-DXA and CT. However, DXA is still not as accurate as CT in, e.g., predicting femoral strength [97].

**Magnetic Resonance Imaging** (MRI) is the most suitable technique method for in vivo 3D geometry modelling since it emits no harmful ionizing radiation. However, although comparable to CT-based geometry models, MRI-based geometry models are not as accurate as CT-based geometry models [151]. The bone microstructure can be effectively imaged by µMRI [53,83]. µMRI-based models are also effective in assessing mechanical properties, but µCT-based models are still more accurate [48,130].

**Microscopy** provides very fine and detailed images featuring the nano and even sub-nano lengthscale bone geometry. However, this technique is invasive and only able to prove 2D geometry [83,132,133,134]. Microscopy-based 3D geometry models can be created when the third dimension is idealized [134], e.g., when a circle is turned into a cylinder. However, creating the third dimension from scratch is not considered subject-specific.

**Remark** **12.** *Very few works in the literature compare two medical imaging techniques to the same bone sample, e.g., [56,151], see column I of Table 2.*

**Remark** **13.** *Scanning and accurately modelling macroscale bone geometry through medical imaging techniques is a mature field of research. However, though HR-pCT, µCT and µMRI allow the determination of bone microscale geometry with some accuracy, techniques capable of accessing non-macro lengthscales must be improved.*

Analytical fracture analysis of complex bone geometries is currently impossible. Numerical methods, see Section 12, used in engineering for structural analysis (including fracture) require discretization of the spatial domain, i.e., a mesh.

**Remark** **14** (Mesh Generation). *The reviewed literature presents two mesh generation procedures for patient-specific bone geometry: (* ***1.*** *) Voxel-based meshing defines the mesh contour as the voxel contour [74,83,168,169]. Each voxel turns into a hexahedron-shaped volume, i.e., cube or rectangular cuboid. This type of mesh generation requires no material mapping strategy, see Section 10. However, it may exhibit locations (corners) where stress is concentrated and can only accurately represent the surface of the bone geometry when the mesh is sufficiently fine. Very fine meshes increase the number of nodes and sub-domains and are thus computationally more expensive; (* ***2.*** *) Geometry-based meshing defines the mesh contour based on the surface of the geometry model [119,168]. It requires a material mapping strategy, see Section 10. Geometry-based meshing is difficult to implement computationally, but several commercial software packages (Ansys, Abaqus, Hypermesh, Gmesh, et cetera) already provide it.*

Completion of the following procedures $9A_{1}$→ $9A_{5}$, see Figure 1 row I, constitute patient-specific bone 3D geometry modelling and discretization:

$9A_{1}$: Scanning—The patient’s bone is scanned by medical imaging equipments, which create a DICOM (Digital Imaging and Communications in Medicine—http://dicom.nema.org/) file. DICOM files contain information on the patient (e.g., age, sex, health condition) in addition to collections of images.

$9A_{2}$: Image segmentation—Medical imaging techniques create images that contain bones, nearby soft tissue and fat. A segmentation must be performed in order to separate bone from non-bone tissues. This can be done manually or automatically [170] by using domestic algorithms or software packages, e.g., InVesalius [171], MIMICS, Simpleware and Amira.

$9A_{3}$: Geometry surface—From segmented *DICOM* files, an *.STL* (STereoLithography) file, describing only the surface geometry of the 3D object, i.e., the hip bone, is obtained by using domestic algorithms or software packages, e.g., InVesalius [171], MIMICS, Simpleware and Amira.

$9A_{4}$: 3D solid geometry model—Using domestic algorithms or software packages, e.g., MIMICS, Simpleware or CATIA, the *.STL* file is converted into a *.STEP* (STandard for the Exchange of Product model data) file, which provides a readily-modifiable 3D solid model of the bone.

$9A_{5}$: 3D mesh—A 3D mesh with *n* nodes and *s* sub-domains is created using domestic algorithms or software packages, e.g., ANSYS, Altair HyperMesh, Gmsh or 3ds Max, from the *.STEP* file. The mesh is described by a node matrix, which contains the coordinates of each node, and an incidence matrix, which relates *n* nodes to *s* sub-domains.

The modelling of the patient-specific bone geometry and thereafter creation of 3D bone meshes are a mature field of research [54,172].

## 10. Mechanical Properties Categorization for Computational Bone Models

Computational bone models require spatially-local data on domain geometry and mechanical properties. Such data can be obtained by applying the standard scientific method:

$10A_{1}$: observation *of* and experimentation *on* bone;

$10A_{2}$: identification of pertinent physical characteristics and phenomena;

$10A_{3}$: selection of mathematical descriptions that match the physical characteristics and phenomena identified in step $10A_{2}$.

When performing $10A_{3}$, most of the *continuum mechanics*-based bone models require, as shown in columns 4, 5 and 6 of Table 2, assumptions regarding:

$10B_{1}$: a constitutive equation;

$10B_{2}$: a type of material symmetry;

$10B_{3}$: homogeneity, i.e., the spatial configuration of material and mechanical properties, of $10B_{1}$ and of $10B_{2}$.

**Open Issue** **2.** *Most of the surveyed literature does not provide justification, based on $10A_{1}$ and $10A_{2}$, for their particular $10A_{3}$.* **Open Issue 2** *aims to hint on what the lack of such justification consists of, by presenting some literature on how $10A_{2}$ influences $10A_{3}$. Some of these influences are mathematically described by appropriate choices of $10B_{1}$, $10B_{2}$ and $10B_{3}$.*

*The surveyed literature points to four groups of open issues regarding the influence of $10A_{2}$ on $10A_{3}$. These groups consist of open issues related to: (* ***1.*** *) bone sample geometry, (* ***2.*** *) bone intensive properties, (* ***3.*** *) phenomenological aspects of bone observation/experimentation and (* ***4*** *) patient-specific characteristics that influence issues (* ***1*** *)–(* ***3*** *).*

*(* ***1*** *) Some issues regarding bone sample geometry:*


*OI 10.11*

*Geometric irregularities at the transverse cross-section: contrary to longitudinal geometric irregularities, they contribute significantly to the linearly elastic torsional behaviour of long bones [173,174].*

*OI 10.12*

*Bone aspect ratio: long bone failure may be more dependent on deformation rather than on stress [175].*

*OI 10.13*

*Microstructure: influences the fatigue life of bone [176,177]. The vascular pattern of bone affects its Young’s Modulus [178].*


*(* ***2*** *) Some issues regarding bone intensive properties:*


*OI 10.21*

*Temperature: influences the fatigue life of bone [176,177].*

*OI 10.22*

*Water content: influences the stiffness, strength and toughness of bone [179,180]; the Young’s Modulus of dead (dry) and living (wet) bones tend to be different [178]. Furthermore, viscoelastic [181] properties of bones are also influenced by the water content. The water content of bone is related to the molecular scale, see Section 13.*

*OI 10.23*

*Mineral content: porosity and mineral content influence bone Young’s Modulus [182,183,184]. The mineral content of bone is related to the molecular scale, see Section 13*

*OI 10.24*

*Bone density: exhibits a p-value based highly significant positive correlation with bone fatigue life [176,177]. Furthermore, density influences bone stiffness and strength [185].*

*OI 10.25*

*Porosity: alongside bone mineral content, influences bone Young’s Modulus [182].*


*(* ***3*** *) Some issues regarding bone phenomenological aspects when under observation/experimentation:*


*OI 10.31*

*Strain-rate: is directly proportional to bone Young’s Modulus under tension and under compression [186] and influences bone compressive strength [187].*

*OI 10.32*

*Loading condition: [178] presents a comparison of bone Young’s Modulus for femur and tibia under tension, compression and bending. Experiments performed by [188] exhibited the same mechanical properties for tension and compression in bone.*

*OI 10.33*

*Stress duration: influences bone Young’s Modulus in a phenomenon labelled elastic after-effect [178].*

*OI 10.34*

*Cyclic loading frequency: influences bone Young’s Modulus [189].*

*OI 10.35*

*Stress amplitude: influences the fatigue life of bone [176,177].*


*(* ***4*** *) Some issues regarding patient-specific bone characteristics:*


*OI 10.41*

*Patient age: affects ultimate tensile strength, elastic modulus, maximum deformation, and Brinell hardness [190] and the bone structure in such a way that increases its fracture risk [191].*

*OI 10.42*

*Diseases: affect the rate of bone remodelling, see Remark 19, and consequently the percentage of bone mineral content (OI 10.23) and BMD distribution, i.e., the mechanical properties of bone [192,193,194]. Fracture risk analysis in unhealthy, e.g., metastatic, bones is currently even less accurate than fracture risk analysis in healthy bones [195].*

*OI 10.43*

*Nutrition: a well-balanced diet (including plant-based diets [196,197,198,199,200]) alongside an adequate intake of Calcium and Vitamin D (sunlight exposure time) may reduce osteoporosis-induced fracture risk and hospital costs [196,201].*

*OI 10.44*

*Physical activity: increases not only quality of life [202,203], but also BMD and bone mechanical properties values [204]. Furthermore, regular exercise enhances bone mass and strength, and reduces bone fracture risk [205].*


A comprehensive review on the relationships between physical, geometrical and mechanical properties of bone was made by ref. [185].

The physical assumptions underlying the conclusion of an experiment may imply an inaccurate mathematical description. For instance, consider a bibliographical reference that states an issue of Young’s Modulus inhomogeneity; this issue may be readily solved using a numerical method, see Section 12, that considers a value of Young’s Modulus that is specific to each discretized unit (or subdomain); perhaps the experiment calculated an equivalent numerical value for Young’s Modulus uniformly distributed in the bone. If a CT were to be used, the conclusions on the same sample would be different.

A mathematical description, despite not explicitly considering all bone physics at the experimental conclusions, can still agree with the experiment. Consider an experiment. A material exhibits properties X and Z of different categories. It may be the case that a change in Z is almost totally reflected as a change in X alone. If X is already considered in the current mathematical description, it may not be necessary to consider any mathematics for Z. The converse can also be true, that is: if, for the same value of X, the outcome of the same experiment is considerably different, it may be the case that Z must be included in the mathematical description.

For instance, if nutrition (*OI 10.43*), diseases (*OI 10.42*), patient age (*OI 10.41*) and density (*OI 10.24*) have their effects fully captured by the knowledge of, e.g., bone mineral content (*OI 10.23*), it is reasonable to assume that these variables are unlikely to appear in an accurate mathematical description. To consider only bone mineral content would suffice for an accurate mathematical description.

The ultimate goal of studying bone fracture is to predict fracture propagation at the macro scale, a task highly dependent on $10B_{1}$–$10B_{3}$. The choice of $10B_{1}$–$10B_{3}$ must be guided by what is observed in experiments. The surveyed literature indicates that $10A_{1}$ implies several mechanical characteristics of bone that are, in a purely macroscopic continuum model, not readily accounted for. Thus, specific models are needed for these mechanical characteristics and their lengthscales. For instance, it is impossible to go straight from the macroscale to the molecular scale. These specific models need to be integrated, from which is known that a non-multiscale approach is incomplete. From the multiscale approach, it is possible to accurately predict fracture propagation at the macro scale through an appropriate choice of $10B_{1}$–$10B_{3}$, see Section 14. These choices can not be accurately made without proper knowledge of all lengthscales, see Section 13. Then it is possible to know if it is a matter of tuning the macroscale model or modeling these different physics someplace else. Multiscale is a consequence of experimental observation.

**Remark** **15.** (Insufficiency of Biological Considerations). *Biology, e.g., using microscopy, provides only the conceptual framework for the material categorization, i.e., for the selection of appropriate $10B_{1}$, $10B_{2}$ and $10B_{3}$. It is through engineering and physics experiments that quantitative mechanical properties are more realistic estimated.*

### 10.1. Constitutive Equation

Calculating stresses and strains over solid bodies using *continuum mechanics*-based bone models requires at least one equation correlating stresses and strains, i.e., a constitutive equation, see Appendix C. There are infinite possible materials, each material described by one or more constitutive equations [206]. Currently known constitutive equations enable simulation-based design of robust materials, e.g., for aircraft. Strain-displacement and motion equations are independent of the material properties, see Figure 1 and Section 12.

**Open Issue** **3.** *Currently known constitutive equations, regarding the mechanical behaviour of living tissues to different loading conditions, do not (or do not accurately) account for, e.g., rapid changes of living tissues over time, e.g., bone remodelling, see Remark 19. Possible dependence on still unknown mechanical properties must be studied.*

This paper found eight constitutive equations used for the creation of computational bone models, listed in column 4 of Table 2: elastic, plastic, viscoelastic and -plastic, poroelastic and -plastic, elastic- and plastic-damageable materials.

**Elastic materials**, see Appendix C, can be either **C**auchy-**L**inear-**E**lastic (CLE) or **C**auchy-**N**on**L**inear-**E**lastic (CNLE), see Figure 1 row VIII. **CLE-materials** display spring-like behaviour according to Hooke’s law: T(x(t),t)=CE(X(t),t). A CLE-material may not comply to Hooke’s Law when there are unknown contributions to the stifness tensor C that are implicitly, but not explicitly, dependent on the strain tensor E. Most of the surveyed literature, as seen in Table 2 column 4, assume that bone is an elastic material; all literature in Table 2(4A) assumes bone complies to Hooke’s Law. Though many materials can be accurately modelled as CLE, the literature on bone mechanical properties rarely reports experimental verifications of CLE-behaviour in bones. **CNLE-materials** are usually modelled by constitutive equations that correlate stress and strain-energy: T≡T(x(t),t,W(E)). Non-linear stress-strain correlations may be linearized into affine approximations [207], which are still not linear correlations. Though none of the surveyed literature reports bone to be CNLE, human soft tissue, also present at muscle-bone connection sites, displays Green-elastic (hyperelastic) behaviour [125,208]. For exhibiting *quasi*-brittle fracture in experiments, bone is sometimes assumed to be a CLE-material [112], e.g., strain measurements performed by [113] have shown this to be a reasonable assumption for femurs. Furthermore, ref. [129,209] assume that the proximal femur behaves as a CLE-material up to fracture, i.e., that the post-yield behaviour, i.e., the plastic behaviour, can be neglected.

**Remark** **16.** (**Linear Material** Terminology). *A material is labelled linear, e.g., Hookean, if it can be accurately modelled by a constitutive equation that exhibits a linear relationship between stress T and strain E. Though not all elastic materials are linear, e.g., Green-elastic materials, only elastic materials may be labelled linear. Viscoelastic materials are, misleadingly, labelled linear materials [210,211] even though they are modelled by a constitutive equation that exhibits a linear relationship between stress T and strain-rate $\dot{E}$ instead of between stress T and strain E. Figure 1 row X classifies materials into* ***L**inear-**E**lastic (LE) and* ***N**on-(**L**inear-**E**lastic) (NLE). Emphasis on the subtle distinction between NLE- and CNLE-materials: the first refers to the set of all materials excluding the LE-materials, the latter refers to the set of all elastic materials excluding CLE-materials.*

Bone can be considered an LE-material for several purposes [212]. However, it is expected that for bone fracture purposes, more “complete” constitutive equations that account for the nonelastic behaviour of bone, may improve the model accuracy and thus the accuracy in predicting fractures.

**Plastic materials**, see Appendix C, feature one or more particles that do not return to their unstressed spatial position after unloaded, thus exhibiting long-term memory of previous stresses and strains. Among the NLE constitutive equations, plastic constitutive equations (or elastic–plastic, elastoplastic) are the most frequently used for modelling bone [184,213,214,215,216]. Plastic constitutive equations may accurately predict the failure of vertebrae [217]. Some of the surveyed literature does not explicitly justify the choice of assuming bone as a plastic material [33]. Nevertheless, the entanglement of different molecules that compose bone may justify its plastic behaviour [218].

**Remark** **17.** (Elastic-Plastic Materials). *No physical material is exclusively elastic or exclusively plastic. The perceived material behaviour depends on experimental setups and local conditions. A certain constitutive equation may better fit the numerical values of the experimentally measured deformations. All materials exhibit elasticity and, after reaching the Yield Stress, plasticity. Thus, the term elastic materials refers to pure elastic materials. The higher the ratio between the Yield Strain and the Ultimate Strain, the higher is the degree of elasticity E of a material; the degree of plasticity is P=1−E. Thus, all plastic materials are, in fact,* ***Elastic-Plastic**, or elastoplastic, materials.*

**Elasticity** and **Plasticity** are modelled by stress-strain constitutive equations. As stated in Remark 3, constitutive equations are not limited to stresses and strains. Constitutive equations can be systems of equations accounting for several phenomena affecting the stress-strain relationship. This papaer presents three such phenomena: **Viscosity**, **Porosity** and **Damageability**.

**Elastic-Viscous materials**, or viscoelastic materials, see Appendix C, exhibit stresses dependent on strain-rate: $T\equiv T(x\left(t\right),t,E,\dot{E})$. Other physical phenomena of viscous materials include stress-strain hysteresis, creep and stress relaxation [219] (p. 436), [220]. Phenomena identified by [178], who studies only aspects $10A_{1}$, $10A_{2}$ were interpreted by [221] as implying that a viscoelastic constitutive equation was an accurate mathematical model for the execution of step $10A_{3}$. Usage of viscoelastic constitutive equations may also be justified by the fact that bone mass is ≈30% collagen, see Section 13.1, which has been experimentally characterized as viscoelastic [206,222,223]. It has been experimentally verified that biological soft-tissue, which is mostly composed of collagen, can be accurately modelled by the Voigt, Maxwell and Kelvin viscoelastic constitutive equations, see [206,224,225] and references therein.

A recent study by [226] showed that boneviscoelasticity is affected by the composition of the molecular scale. Viscoelastic parameters measured at the macroscale may not be directly related to viscoelastic parameters measured at lower-scales, see Definition 9. A new microscopic viscous-hyperelastic constitutive equation for human trabecular bone based on depth-sensing indentation tests was presented by [227].

**Plastic-Viscous materials**, or viscoplastic materials, see Appendix C, are plastic materials that exhibit post-yield strain-rate dependency, which has been experimentally verified at the macroscale [228,229,230,231]. Still, few works ventured to model bone as a viscoplastic material.

**Highlight** **6.** *An Elastic-Plastic-Viscous constitutive equation for the analysis of trabecular bone under compression is presented by [232].*

The water content within bone may also explain its viscous (both viscoelastic and viscoplastic) behaviour [233].

**Definition** **4** (Porous Material). *Porous materials consist of a solid body topologically defined over a simply connected spatial domain whose convex hull features non-solid gaps. The non-solid gaps are known as pores.*

**Elastic-Porous materials**, or poroelastic materials, see Appendix C, in which fluid flows through porous elastic solids, are modelled by equations from the theories: *of* elasticity, *of* viscous fluid flow and *of* fluid flow through porous media, see [222,234] and references therein. When devising a multiscale poroelastic cortical bone model, ref. [144] found that the fluid flow influences the stiffness of bone. A constitutive equation accounting for the pressure both in the *material pores* $10C_{1}$ and over interconnected *fluid compartments* $10C_{2}$ within a porous solid is studied in [235]; in bone, $10C_{1}$ may refer to the collagen-water-hydroxyapatite-lattice lengthscale, see Section 13, and $10C_{2}$ may refer to the bone marrow-filled intertrabecular pores, see Section 13.

**Plastic-Porous materials**, or poroplastic materials, see Appendix C, in which fluid flows through porous plastic solids, are modelled by equations from the theories: *of* plasticity, *of* viscous fluid flow and *of* fluid flow through porous media. From a generic poroplastic model for binary mixtures, where the mixture may be assumed as consisting of solid bone and biomaterial, ref. [236] estimated the yield stress associated with the outset of remodelling, see Remark 19

**Definition** **5** (Damageable Materials). *In this paper’s terminology, Damageable Materials refers to materials accurately modelled by damage-accounting constitutive equations, see Appendix C—Damage Mechanics. Such constitutive equations are obtained by modifying any non-damage-accounting constitutive equation; these modifications include, in a known constitutive equation, a damage variable which is a mathematical representation for an ensemble of microdefects in the spatial domain of the material [237].*

**Elastic-Damageable materials** have been considered by [60,76].

**Plastic-Damageable materials** have been considered by [54,56,64,73,75,80,124].

Other materials, modelled using constitutive equations combining Elastic-Plastic-Viscous-Porous-Damageable materials are possible. For instance, by devising a trabecular bone model with both *poro-* and viscoelastic constitutive equations, [238] argued that, at certain lengthscales, viscoelasticity, not poroelasticity, accounts for almost the entirety of the “total stress” over a cubic bone sample.

**Remark** **18.** *The specialized experimental literature shows that bone may be accurately modelled as an elastic-plastic-viscous-porous-damageable material. Indeed, it is reasonable to assume that bone (and any other material) exhibits a -as complex as possible/accounting for all variables- mechanical behaviour. However, when modelling bone fracture, it is not necessary to account for all possible variables to reach accurate fracture predictions. For example, metals are in reality anisotropic, see Section 10.2, but isotropic computational models of metals exhibit accurate predictions used by design and structural engineers. It is thus important that physicists and engineers find out which variables and which constitutive equation satisfactorily models and predicts bone fracture.*

**Remark** **19** (Bone Remodelling). *In brief, Wolff’s law (originally in German, Das Gesetz der Transformation der Knochen [239] enunciates that: living bones tend to become stiffer and denser when periodically loaded; on the other hand, when not periodically loaded, bones shrink and become more fracture-susceptible. Wolff’s law is best described by bone remodelling [240], which is basically characterized by two processes [241,242,243]: (* ***1.*** *) bone resorption, i.e., bone tissue erosion by osteoclasts; (* ***2.*** *) bone formation, i.e., bone synthesis by osteoblasts. Osteoporosis and several other bone diseases are a consequence of bone remodelling malfunction [244,245], i.e., higher ration of bone resorption in comparison with bone formation.*

Wolff’s law and bone remodelling explain, for instance, a phenomenon known as stress shielding, see Definition 6, and also why astronauts exhibit thinner bones. In space, astronauts are exposed to lower levels of gravity than on earth, meaning that their bones will be subjected to lower stresses.

**Definition** **6** (Stress Shielding). *Bone implants are usually made of materials that exhibit much higher stiffness (and general mechanical properties) than bones. The stresses applied on a bone in contact with an implant tend to be shielded from that bone by the implant. This bone tends, therefore, to become less dense and stiff, as described by Wolff’s law, see Remark 19. This loss of density and stiffness caused by much stiffer bone implants is labeled stress shielding [246].*

Furthermore, Wolff’s research on femoral heads found out that trabecular bone adapts its orientation in the direction of applied forces, seeking an optimal inner structure with minimum stress concentration. This change in geometry follows the forces acting within the trabeculae and is mathematically describable, i.e., it behaves following mathematical laws [147,243,247,248].

### 10.2. Material Symmetry

Material symmetry regarding mechanical properties [249] (p. 84), defined for LE-materials only, may be assumed as being of one type out of eight possible types [222,250]: isotropic, cubic, transversally isotropic, tetragonal, trigonal, orthotropic, monoclinic and triclinic. Materials featuring symmetry types 2–7 are labeled anisotropic. Though it is not impossible for a NLE-material to feature a type of material symmetry, no material symmetry categorization for such materials exists, as seen in Figure 1. Every possible material is either LE or NLE, see Remark 16.

The survey found 3 main types of material symmetry used to devise LE computational bone models; they are listed in column 5 of Table 2 and are further discussed.

**Isotropic materials**, see Appendix C, are the most implemented material symmetry among the surveyed literature, see Table 2(5A). Isotropic materials are easier to implement than anisotropic materials (they possess only two independent constants out of twenty-one possible, triclinic). Patient-specific, e.g., QCT-based, estimation of anisotropic material symmetry is still a non-mature field of research [31,36]. This might be another reason why isotropic materials are more often implemented, especially among patient-specific computational bone models.

**Open Issue** **4** (Isotropy Assumption). *Depending on the conditioning of the elastic stiffness matrix, a theoretically anisotropic bone can be accurately represented as an isotropic material, e.g., small differences between stresses and displacements calculated assuming isotropic and orthotropic patient-specific mechanical properties have been presented by [44]. Anisotropic models, when compared with isotropic models, sometimes exhibit a minimum effect on the correlation between macroscale analysis and experiments [81,115,127,251,252], sometimes exhibit relevant improvements.*

**Transversal Isotropic materials**, see Appendix C, are the most implemented anisotropic material symmetry among the surveyed literature, see Table 2(5C). Bone exhibited experimental transversely isotropic material symmetry in some works [233,253,254]. Recent works modelled bone as a transversally isotropic material [220,255].

**Orthotropic materials**, see Appendix C, are considered to best describe bone material symmetry. Bone exhibits orthotropic material behaviour in many works, e.g., [44,212,256]. However, small differences between stresses and displacements calculated assuming isotropic and orthotropic patient-specific mechanical properties have been found, e.g., by [44].

Though bone consistently seems to be orthotropic, its LE-symmetry is subject-specific, and thus patient-specific [222]. The same bone from different individuals may present different material symmetry.

**Triclinic materials**, or general anisotropic materials, and other types of material symmetry, see Appendix C, and their application to model bone is still a non-mature field of research. In vitro experiments have not shown such behaviours. That is mainly because triclinic material symmetry could not be experimentally measured and identified [212]; triclinic symmetry could only be assumed. Later, however, experimental methodologies for determination of all triclinic symmetry parameters was presented [257,258].

Few works are found in the literature applying a material symmetry not shown in column 5 of Table 2. Although no definite statement can be made on the real in vivo behaviour of bone, in vitro bone experiments exhibit anisotropic mechanical properties [221,259]. Yet, the great majority of the literature assumed bone and its components (e.g., hydroxyapatite, collagen) to be isotropic, see Table 2.

Modelling bone as an anisotropic material may improve fracture risk predictions, but anisotropy might not be obtained from medical imaging, i.e., may not be obtained from patient-specific methods [36].

### 10.3. Homogeneity Regarding Constitutive Equation and Anisotropy

Solid bodies, in regard to the spatial distribution of their mechanical properties, can either be homogeneous or inhomogeneous, see Appendix C. Computational bone models listed in column 6 of Table 2 assumed bone to be at times homogeneous, at times inhomogeneous.

**Homogeneous materials**, see Appendix C, feature, at any arbitrary pair of points within their spatial domain, mechanical properties of the same numerical value. Though some materials can be accurately modelled as homogeneous, no real-world material fits such description. Computer implementation of a homogeneous material is a mature field of research.

**Inhomogeneous materials**, see Appendix C, feature, at any arbitrary pair of points within their spatial domain, mechanical properties that are not necessarily of the same numerical value. Estimation of inhomogeneous mechanical properties from medical imaging-based geometry models is straightforward and has been performed by many of the references in Table 2(6C).

**Open Issue** **5.** *Devising standardized material tests for the obtention of experimental measurements bone mechanical properties remains an open issue. The multiscale structure of bone makes mechanical properties both lengthscale- and bone site-dependent, see Section 13.*

Patient-specific inhomogeneous bones are most commonly modelled by splitting the spatial domain into smaller homogeneous subdomains and assigning specific mechanical properties to each subdomain. These subdomain-specific mechanical properties can be computed from medical imaging data, e.g., CT. The definition of subdomains requires domain discretization, see Remark 14 and $9A_{5}$.

**Remark** **20** (A General Remark on the Physics of Bone Modelling). *Though outside the scope of this review, some scarcely studied phenomena include: the effect of fiber orientation; the rate of loading; the velocity of impact; the spatial distribution of calcium; the dependence of Young’s Modulus and damping on bone site; piezoelectricity; bone aspect ratio; stiffness reduction after the initial formation of small cracks [260]; the decrease of stress concentration factor around holes in the presence of couple-stress effects [261]; creep effects [262]; application of micropolar theory and couple stress theory [263]; drying and re-wetting effects [264]; osteonal microstructure and cortical porosity differences that may be adaptations related to regional differences in strain mode and/or strain magnitude [265]; disparity in mechanical properties of compact bone in tension vs. compression; the influence of bone integrity [266]; work of fracture [267]; Terzaghi’s effective stress [268].*

### 10.4. Patient-Specific Mechanical Properties

Medical imaging techniques have often been used to estimate bone patient-specific mechanical properties, see Table 2(7C). CT is the most used technique for assigning patient-specific homogeneous, LE, isotropic properties to sub-domains of a mesh, see Table 2(1A), [38,97,98,111,112,118,119,269,270,271,272,273].

**Figure 2:**
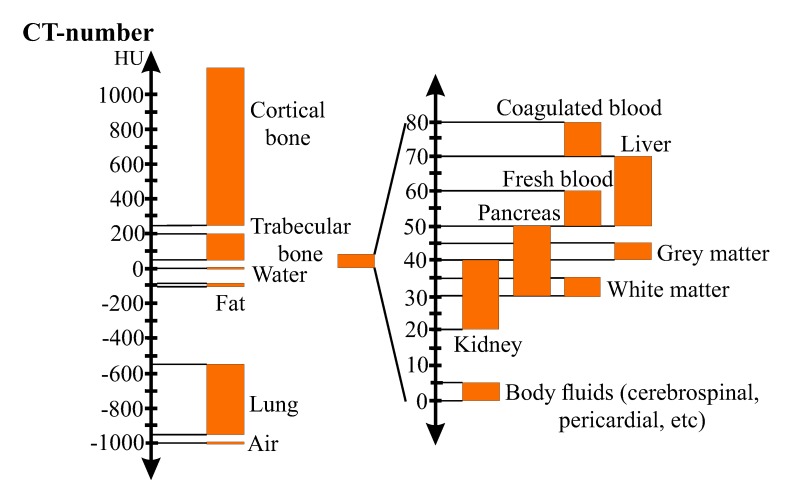
Hounsfield-Scale for different kinds of tissues (adapted from *©* Institut für Anatomie, Universität Bern (https://elearning.medizin.unibe.ch/morphomed/radioanatomie/ct-mrt-des-rumpfs/ct-mrt-einf%C3%BChrung/hounsfield-skala).

CT cross-sectional images (CT-images or CT-slices) are created by X-ray tubes and detectors, which rapidly rotate around the patient’s body while the patient is slowly moved through the ring-shaped CT-equipment. The emitted radiation penetrates the patient’s body and is either totally or partially absorbed. The detectors receive the residual radiation and send electrical signals to computers. Calculations generate cross-sectional images of the patient’s body. Each CT-image is interpreted by the computer as a pixel-matrix. Pixels (picture elements) are the elements of the matrix (3D pixels are labelled voxels, i.e., volume elements or volumetric pixels). Each pixel is assigned a linear attenuation coefficient, which is converted into Hounsfield Units (HU) [274] by

(1) $$CT_{\mathrm{num}}\left(\mu_{\mathrm{tissue}}\right)=1000\frac{\left(\mu_{\mathrm{tissue}}-\mu_{H_{2}O}\right)}{\mu_{H_{2}O}}\left[HU\right]\;\mathrm{or}\;CT_{\mathrm{num}}\left(\mu_{\mathrm{tissue}}\right)=1000\frac{\left(\mu_{\mathrm{tissue}}-\mu_{H_{2}O}\right)}{\left(\mu_{H_{2}O}-\mu_{\mathrm{air}}\right)}\left[HU\right]$$

where $CT_{\mathrm{num}}$ indicates the CT-number, i.e., grayscaled pixel-value given in HU; and $\mu_{\mathrm{tissue}}$ and $\mu_{H_{2}O}$ represent the attenuation coefficients of the tissue (pixel) and water, respectively. The Hounsfield Unit was created such that the CT-number of water and air are set to 0 and −1024 HU, respectively. It provides a more tangible reference for values seen in the grayscaled CT-images. Figure 2 illustrates the Hounsfield-scale for different kinds of biological tissues.

When a calibration phantom is scanned with the bone, HU-values can be converted into volumetric bone mineral density (vBMD), also labelled as radiological density or quantitative equivalent CT-density ($\rho_{\mathrm{QCT}}$), using an affine function [36,153,275,276]:

(2) $$\rho_{\mathrm{QCT}}=a\;\cdot \;CT_{\mathrm{num}}\left(HU\right)+b$$

**Remark** **21** (vBMD). *CT is not the only medical imaging technique able to quantify vBMD. MRI-images, though not as suited to estimate bone density as CT-images [150,277], were used to accurately quantify in vivo vBMD of a patella by [278] and to estimate CLE-properties [48,131].*

**Highlight** **7** (Patient-Specific Phantomless Estimation of BMD). *Very recently, a phantomless method of estimating vBMD from HU was proposed by [279].*

In the context of bones, depending on experimental measurements, density can be defined in different ways [280]. The three most relevant density measures in the context of patient-specific material properties estimation are [187,275,276]:

$$\mathrm{Real}\;\mathrm{density}:\rho_{\mathrm{real}}=\frac{\mathrm{wet}\;\mathrm{mass}}{\mathrm{solid}\;\mathrm{volume}};$$

   

$$\mathrm{Apparent}\;\mathrm{density}:\rho_{\mathrm{app}}=\frac{\mathrm{wet}\;\mathrm{mass}}{\mathrm{bulk}\;\mathrm{volume}};$$

   

$$\mathrm{Ash}\;\mathrm{density}:\rho_{\mathrm{ash}}=\frac{\mathrm{ash}\;\mathrm{mass}}{\mathrm{bulk}\;\mathrm{volume}};$$

where bulk volume is the total volume of the solid and non-solid material, $V_{\mathrm{bulk}}=V_{\mathrm{solid}}+V_{\mathrm{non}-\mathrm{solid}.}$ The solid volume is the volume occupied by the solid material only, not including porosity.

The different types of bone density are directly correlated. The literature on the relationships used in the conversion of $\rho_{\mathrm{QCT}}$ measures to $\rho_{\mathrm{ash}}$, $\rho_{\mathrm{app}}$, and $\rho_{\mathrm{real}}$ (or tissue density) as well as the relationships between these densities and the CLE-properties of patient-specific bone was reviewed by [275].

The existence of density-elasticity relationships, i.e., relationships between CT-estimated bone density and LE properties, e.g., Young’s Modulus *Y*, has been empirically studied by [102,118,169,281,282] and the works therein. Density-elasticity relationships are usually represented by a power function and have a great influence on the prediction of the CLE-properties of bone [36,68].

(3) $$Y=k\;\cdot \;\rho_{\mathrm{ash}/\mathrm{app}}^{p}$$

where the coefficients *k* and *p* are experimentally estimated. Though most commonly estimated through experiments, density-elasticity relationships can be determined by inverse computational approaches [36,283,284]. Due to the uniqueness of each bone, there is no density-elasticity relationship that accurately estimates macroscopic mechanical properties for all bones [214,280]. In the hypothesis of [68] that each bone specimen has its individual density-elasticity relationship, bone CLE-properties are assumed to be patient-specific, suggesting that a density-elasticity relationship should be determined for each bone.

According to [102], who compares several density-elasticity relationships, some of the published relationships are unsuitable for strain prediction in bones. However, subject-specific models that used the density-elasticity relationship proposed by [281] showed very good accuracy in predicting strains [102,111]. Relationships proposed by [256,285] showed less accuracy. The density-elasticity relationship proposed by [281] also exhibits good strain and strain-energy predictions in long bones [116] and in shoulders [30].

**Remark** **22** (Bone Mechanical Properties from Continuum Micromechanics). *It is possible to replace purely empirical, CT-based, HU-density-elasticity relationships by other relationships based on continuum micromechanics that consider the micro-morphological features of bone* *within* *each voxel of a CT-image. Such relationships account for voxel-specific: bone structure, vascular porosity and volume fractions of HA, CLG and $H_{2}O$, see Section 13. Usage of such relationships may improve mechanical behaviour prediction [115,140,286,287,288,289,290,291]. Emphasis on [115], the first to consider an inhomogeneous Poisson’s ratio for bone. Continuum micromechanics may be coupled with ultrasonic experiments, instead of being coupled with CT, to estimate the CLE-properties of bone [292].*

The process of assigning homogeneous mechanical properties $10B_{1}$, $10B_{2}$ from medical images onto sub-domains of a 3D-mesh $9A_{5}$ of an inhomogeneous computational bone model, see Definition 2, is labeled *material mapping* [75,98,118,119]. The strategy used to perform a material mapping can have a great impact on the assignment of bone CLE-properties [118,119].

There are several software packages that perform a material mapping. SimpleWare ScanIP and MIMICS are the most consolidated. Several works used the freeware Bonemat to perform material mapping [38,119,120,269,270]. Bonemat was also used as a reference to develop and validate other material mapping strategies tools [97,118,271,272].

**Open Issue** **6.** *CT-images are two-dimensional and CT-voxels are points in space, therefore, a voxel does not contain local anisotropy. However, different techniques and approaches to extract anisotropic mechanical properties from CT-data were proposed in the literature [44,73,74,81,168,221,251,286,293]. Most commonly, different values of k and p, see Equation (3), are given for different directions. The micromechanics-based approach shown in [115,140,286,287,288,289,290,291] does not derives anisotropic properties purely from CT-images, but correlates each CT-voxel to an anisotropic tensor based on the voxel-specific volume fractions of HA, CLG and $H_{2}O$, see Section 13.*

*Yet, as mentioned in Section 10, the influence of anisotropy on the accuracy of the model’s behaviour, as the extraction of anisotropic properties from medical images, is still an open issue [81,251].*

Cortical and trabecular bones exhibit different geometries and mechanical properties, see Section 13, thus, they require different density-elasticity relationships [44,90,92,93,106,115,121]. Further, some works define an upper limit for the Young’s Moduli (or HU values) evaluated using density-elasticity relationships, since external interferences or error by the scanning may occur [44,87,269].

## 11. Mathematical Model of Bone Trauma-Inducing Accident—The Boundary Conditions

BCs for patient-specific fracture simulations should preferably be derived from realistic models of common accidents among the elderly. In a solid mechanics problem, for instance, BCs are usually represented as surface forces and displacements.

**Figure 3:**
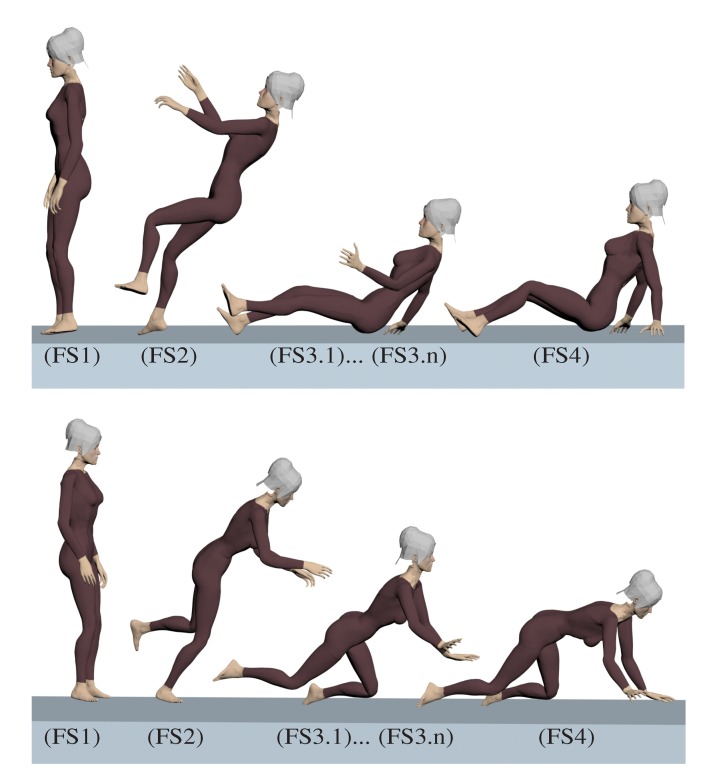
Backwards and forwards fall.

Figure 3 illustrates examples of falls, backwards and forwards, that may originate fragility fractures. The “*” in the accident model box in Figure 1 row II indicates that this stage is represented in Figure 3.

Both backwards and forwards falls can be seen as a sequence of instantaneous motions, illustrated in Figure 3 by Fall Stages (FSs).


**FS 1**
Normal human gait, i.e., walk or run. The individual is in motion through the movements of the legs, e.g., at stance position.
**FS 2**
Tip over, or equilibrium loss. This stage characterizes the fall. The equilibrium loss occurs when the challenge to balance is greater than the ability or strength to stay upright.
**FS 3.1**
1st environment collision. The first collision between the body and a solid surface from the environment, e.g., the floor. It is usually the most intense and fracture-susceptible collision. The first collision is usually followed by a series of other collisions caused by inertial movements. Picture a bouncing ball; the idea is the same. As long as the inertial forces are greater than the ability to stop them, collisions will follow.
**FS 3.2**
>2nd environment collision. The second collision may have one or more contact points, or zones, between the body and environment, e.g., the individual may hit the floor with both hands or with hip and a hand at the same time.
**FS 3.i**
*i*-th environment collision. The i-th collision may have one or more contact points, or zones, between the body and the environment.
**FS 3.n**
*n*-th environment collision. Similar to the second collision, the n-th, and last, collision may have one or more contact points, or zones, between the body and environment. It is often the least fracture-susceptible collision. The first collisions have already absorbed most of the kinetic energy of the fall.
**FS 4**
Final position. Characterizes the accommodation of the body. Here there is only minor motion. There is no more collision with the environment. The individual has already fallen and looks for a rest position. The accommodating motion is not relevant for fracture.

This description of FSs is valid for any bone and any fall. Side-ways falls have not been illustrated in Figure 3, but exhibit similar FSs. Depending on environment obstacles, individual reflex and motor skills, a different bone can first collide with the environment at **FS 3.1**. Furthermore, all instantaneous motion described by FSs can be considered a *quasi*-static equilibrium. Thus, it is reasonable to evaluate the motion equation in a *quasi*-static sense, i.e., equilibrium equation [147,294], see Governing Equation (1).

In Figure 3, the collision happens between the body and the environment. However, most of the surveyed papers simulate bone individually, i.e., external forces are applied directly on the bone; neighbour-tissues are neglected. Forces applied on the exterior part of the body, i.e., on the skin, are not the same as the forces acting directly on the bone at the interior of the body.

**Open Issue** **7** (Body BC vs. Bone BC). *The tissues between the bone and the contact point partially absorb the impact, displaying a damping effect. When transporting BCs from contact points to the boundary of the bone, the energy absorbed by these tissues should be taken into account.*

*Very recently, a model which predicts the fraction of the collision force that is transferred to the boundary of bones was presented by [126]. This model takes into account damping effects due to flooring elements (i.e., carpets), protector devices (if present), all active tissues (muscles) that contract at the instant of impact and all passive soft tissues interposed between the point of impact on the skin and the lateral aspect of the greater trochanter of the femur.*

In general, the simulated bone should always be assumed as the limiting bone, i.e., as the bone that will fracture first. Each kind of fall has its limiting bone. For instance: (**1.**) if you fall on your hand(s), the wrist is the most fracture-susceptible bone; (**2.**) if you fall on your back, the spine is the most fracture-susceptible bone; (**3.**) if you fall on your backside, the hip bone is the most fracture-susceptible bone, (**4.**) if you fall on your knees, the femur is the most fracture-susceptible bone.

Neighbour-bones of the limiting bone are also usually affected by the collision, but less directly and critically. The forces acting on these bones have been already damped by other tissues. Furthermore, between **FS3.1** and **FS3.n** there are multiple collisions with the environment. It may be argued that only the most intense and fracture-susceptible collision should be modelled for being the most relevant one, however, a sequence of many less intense collisions may also lead the bone to fracture.

**Remark** **23** (Multiple Collisions). *No fracture resulting from multiple collisions was considered in the surveyed literature.*

Highlight 8 summarizes the surveyed papers with major contributions and interesting findings on deriving BCs from dynamic models and imposing them onto the bone surface.

**Highlight** **8.** *  A model accounting for fall rate, stochasticity of fall scenarios including fall kinematics, postural reflex and fall impact attenuation conditions was presented by [126].*

*The interaction between body and ground using a mass-spring-damper system and patient-specific variables, e.g., hip soft tissue thickness, body mass index, body height and weight, was modelled by [35,295,296]. Conclusions showed that patient-specific dynamic models can improve the accuracy of hip fracture risk analysis.*

*The influence of loading direction on strength and fracture sites of the proximal femur is presented by [100]. A CT-based FEM, see Section 12, is used to determine loading directions under which the proximal femur is most fracture-susceptible.*

*Patient-specific loading forces acting on the proximal femur during a sideways fall is estimated by [39].*

*Loading conditions mimicking typical sideways falls on the hip are modelled by [41]. Femoral neck internal rotation angles varying from −30° to* 45° *at* 15° *intervals are selected to simulate a range of possible falling configurations.*

*A free library available by http://www.orthoload.com provides a direct approach to estimate force BCs acting in human joints [160].*

*Homogenized yield properties of human femoral trabecular bone are evaluated and compared in [297] by applying kinematic uniform BCs and periodicity-compatible mixed uniform.*

*Using basic principles of kinematics and dynamics, ref. [298] estimates peak impact forces on the greater trochanter in sideways falls from standing height.*

*A comprehensive database of hip contact forces and simultaneously measured gait data for improvements of hip implants is provided by [299].*

*Very recently, ref. [300] studied the influence of BCs on bone fracture assessed using the FEM, see Section 12.*

BCs should very closely imitate in-vivo situations and be as simple as possible so simulations can be experimentally validated [160], see Section 15. The estimation of the forces exerted by muscles, ligaments constraints, and joint reactions is still a major scientific challenge [111]. BCs strongly influence elastic-plastic mechanical properties of heterogeneous materials estimated by homogenization techniques, see Section 14, [297], and are of major relevance for the accuracy of fracture simulation.

The main goal of determining appropriate and realistic BCs from fall models is to calculate stress and strain fields within the bone geometry, which can be further related to fracture.

In Table 2(8A), many of the surveyed references estimate BCs from very simple accident models. A specific force is simply and directly applied on the femur’s head and said to be the representation of a side-ways fall or stance position [91,105,106,108,109,110,111,112,121,122,129].

## 12. Simulating Bone Fracture

Figure 1 illustrates the PDEs that describe (or “govern”, see Remark 24) the solid continuum mechanics. Motion and strain-displacement equations are briefly discussed in this section. Constitutive equations are discussed in Section 10.

**Remark** **24** (Governing Equation). *Governing equations are mathematical equations that describe phenomena, natural or otherwise. Solving governing equations provides the values of previously unknown dependent variables based on changes of known independent variables and on the numerical value of relevant physical constants.*

**Governing Equation** **1** (Motion Equation). *The macroscopic geometry defines the spatial macrodomain $D\in R^{3}$. A patient-specific macroscopic geometry is devised through a mesh, see Remark 14. The motion equation, see Appendix C, is the governing equation of D and stems from the balance of linear momentum; it can either describe accelerated motion or an equilibrium configuration, see Figure 1 row* V.

*The motion equation represents an equilibrium and is labeled equilibrium equation when the inertial term is neglected [147,294], i.e., $\ddot{x}\left(t\right)\approx 0$, and hence ∇·T(x(t),t)+b(x(t),t)=0. This is appropriate when inertial forces in Figure 3 are much smaller than body and surface forces. For instance, a fall from standing height may be considered a quasi-static equilibrium [71,105,147,294,301].*

**Governing Equation** **2** (Strain-Displacement Equation). *The strain-displacement equation, see Appendix C, relates the strain tensor E(u(X(t),t),X(t),t) and the displacement vector field u(X(t),t). When only relatively small strains, displacements and rotations are considered, the non-linear, i.e., second-order terms, of the strain-displacement equation, i.e., $\nabla u\left(X\left(t\right),t\right){\nabla u}^{T}\left(X\left(t\right),t\right)$, are neglected, and hence $E\left(u\left(X\left(t\right),t\right),X\left(t\right),t\right)=\frac{1}{2}\left(\nabla u\left(X\left(t\right),t\right)+{\nabla u}^{T}\left(X\left(t\right),t\right)\right)$, see Figure 1 row* V.

**Governing Equation** **3** (Compatibility Equation). *The compatibility equation guarantees that there is a single-valued displacement vector field u(X(t),t) associated to each point of the spatial domain D. The compatibility equation is needed in a continuum solid mechanics problem only when strains E(u(X(t),t),X(t),t) are given as inputs. In bone fracture analysis, however, displacements u(X(t),t) are given as inputs, i.e., as BCs, see Figure 1 rows IV to VI. Thus, the compatibility equation is usually not necessary in the algorithm for a computer simulation.*

Different forms and assumptions of the governing PDEs may have specific terminologies that are commonly misused by the literature on bone fracture and continuum mechanics.

**Remark** **25** (Misleading Term—Nonlinear Analysis). *Terms such as nonlinear analysis may be misleading. For instance, [99,101,131] speak of non-linear analysis, but do not specify the type or source of non-linearity. There are mainly four types/sources of non-linearity in a solid continuum mechanics problem [302] (p. 85), [303] (p. 8): (* ***1.*** *) material nonlinearity, i.e., when an NLE constitutive equation is used, see Remark 16; (* ***2.*** *) geometric nonlinearity, i.e., the strain-displacement equation does not include the second-order term, see Governing Equation (2); (* ***3.*** *) kinematic non-linearity, i.e., when the displacement BCs depend on the deformations of the structure; and (* ***4.*** *) force nonlinearity, i.e., when the applied forces depend on the deformation of the structure.*

The PDEs that describe deformations in solid continuum mechanics problems cannot be solved analytically for complex geometries, e.g., bones. Numerical methods capable of solving PDEs through approximations are required. Three main numerical methods can be applied in solid continuum mechanics:

The **Finite Element Method** (FEM) subdivides the spatial domain into subdomains (or elements) and approximates the governing equations by traditional variational methods over each subdomain [303]. The FEM is by far the most used numerical method in the bone fracture literature [148,160,304]. Most probably because there are many commercial software with friendly user interfaces that facilitate its operation, and because the FEM is a mature field of research which has been optimized for several applications. For instance, FAIM, a finite element solver optimized for solid mechanics simulations of bone, was developed by [305,306].

The **Boundary Element Method** (BEM) requires discretization of the boundary only and, for this reason, usually requires a smaller number of DOF than the FEM to achieve accurate results [307,308]. A discretization of the spatial domain into subdomains, commonly labelled subregions by the literature on BEM, is required when the analyzed material is inhomogeneous [307,309], see Section 10. The BEM has been scarcely used in the field of bone fracture. However, some works have used the BEM for bone remodelling simulation [310,311,312,313,314,315].

The **Finite Difference Method** (FDM) is simple in formulation, but exhibits some difficulties in modelling complex geometries and, for this reason, has been scarcely used for solid mechanics problems in recent years [316,317]. FDM was used by [318] to simulate bone remodelling, see Definition 19.

Other numerical methods are also available, e.g., method of characteristics, finite volume method, et cetera; these are outside our paper because, to the best of the authors’ knowledge, they have not yet been applied to bones.

**Open Issue** **8** (Exploring BEM and FDM). *The BEM and the FDM have been scarcely used by the literature on bone fracture. The BEM is, however, particularly recommended for fracture mechanics problems [309]. The FDM succeeded in fluid dynamics and is mostly used when studying fluid and wave propagation within bones [319,320].*

**Remark** **26** (Inputs for Numerical Methods). *A mesh covering the geometry (Remark 14), mechanical properties $10B_{1}$–$10B_{3}$ and BCs, see Section 11. The non-mathematical reader should know that all numerical solution procedures share these same inputs.*

The primary goal of fracture simulation is to evaluate strain and stress fields and to associate them with failure criteria. A recent review made by [148] has shown that stress- and strain-based failure criteria may improve the prediction of fractures [112,321].

There are several different ways of approaching fracture mechanics problems [322,323,324,325]. **Linear Elastic Fracture Mechanics** (LEFM), the classical and mature cracking process mathematical model, is restricted to elastic materials. Though largely applied, LEFM is not the most appropriate approach to describe crack propagation in bones [326]. **Elastic-Plastic Fracture Mechanics** (EPFM), though more recommended for materials exhibiting large plastic zones (of the same order of magnitude as the crack size) at the crack tip, has been less successful than LEFM in predicting fracture when large yielding prevails [324]. The **Cohesive Zone Model** (CZM) is based on considering fracture separation occurring at an extended zone ahead of the crack tip (also labelled “cohesive zone”). Two reasons make the CZM superior to LEFM for bone fracture analysis: (**1.**) Bone fracture experimental data analysis performed by [327] demonstrated the need for a nonlinear model considering a spatial stress distribution at the fracture zone. (**2.**) Unlike the LEFM, the CZM can remove stress singularities ahead of the crack tip; i.e., ahead of the furthest extent of damage [143]. Furthermore, both LEFM and EPFM require a pre-existing initial crack, whereas the CZM can be modelled at the interface between continuum elements (spatial sub-domains) [328].

**Remark** **27** (Animal Bone Modelling and Simulation). *Parallel to the work conducted on modelling human bone, there have been efforts to model bone of several animals. Studies range from small animals such as mice [287,329,330,331,332,333,334,335,336], rats [288,337,338,339,340,341,342,343] and zebrafish [344], to medium-sized animals such as dogs [345], as well as large animals such as pigs [346,347,348,349,350,351,352], sheep [353,354,355], bovine [220,356,357,358,359,360,361,362,363] and horses [273,364].*

*As it is done with human bone, subject-specific animal bone geometry models are created from CT-data [345,365], see Section 9. The influence of CT resolution was investigated by [357,363,366]. Some works used  µCT to create high-resolution animal bone geometry models [287,344,365,366].*

*There are not many dynamic models of animal motion [364,365,367]. Due to this apparent lack of interest in animal motion simulation, most animal bone simulations use BCs representative of experiments, see Table 2(8C), e.g., compression [340], 3-point bending tests [357,361] and 4-point bending tests [349]. Thus, simulations of clinically relevant situations in animal models is not those of human models.*

*Fabricating specimens, see Definition 7, from animal whole bone samples compromises experiment reliability, even more so in the case of small animals. Thus, most experiments are made on whole bones. It is difficult to hold small animal bone samples, e.g., mice bone [336], in a fixed position during experiments due to the presence of asymmetrical loadings, e.g., twisting of the sample at the areas touching experimental apparatus can easily occur [330,335].*

*Bones, human and animal alike, are modelled and simulated using the same techniques to: acquire bone geometry, see Section 9; estimate mechanical properties, see Section 10; use equations based on continuum mechanics, see Section 4; solve said equations using numerical methods, see Section 12. Furthermore, models of mechanical and reconstructive properties of animal bones, e.g., osseointegration, bone ingrowth and bone marrow reconstruction were investigated by [333,337,339,341,347,359,368].*

*Research directives within the field of animal bone modelling and analysis include $12A_{1}$–$12A_{3}$.*

*$12A_{1}$: Usage of animal bone experimental data to estimate mechanical properties [220,287,288,330,331,340,342,347,363,369] and conception of animal bone failure models [331,338,349,351,356,358,362] that may be similar to human bone failure models [342,344,370]. This directive includes studies in which pathological changes to the skeleton are purposefully induced in test animals by genetic manipulation [371] or malnutrition [332,338,354] in order to create models to study osteoporosis [338,354].*

*$12A_{2}$: Biocompatibility evaluation, via in vivo experiments, of implant materials [339,355,356,360,372,373], implant designs [352,374], and surgical techniques [353], with the host tissue. These evaluations, are first performed on animal tissues as a stepping stone towards application in human tissues.*

*$12A_{3}$: Conception of models that: explain animal bone mechanical behaviour [369,375]; compute key variables of animal implant design [376]; and help in designing treatment procedures [345,348,350]. These studies mostly focus on domestic and farm animals.*

**Definition** **7** (Specimen). *A specimen is a standardized material sample meant to represent larger quantities of the same material and built for controlled laboratory experiments.*

## 13. The Multiscale Structure of Bone

To improve bone fracture risk analysis, more accurate simulations are required. To perform more accurate simulations, more realistic models are needed. To create more realistic models, a deep knowledge of the multiscale structure of bone is required. The multiscale structure of bone refers to the complex network of different physical structures and mechanical properties present throughout bone tissue down to the atomic scale, where fracture ultimately originates.

The geometry and mechanical properties $10B_{1}$–$10B_{3}$ of each lengthscale of bone are influenced by the geometry and mechanical properties of lower-scales, see Definition 9. Similarly, the geometry and mechanical properties of the lowest possible continuum lengthscale is affected by molecular features, e.g., by the arrangement and distribution of the molecular structure. Geometry, mechanical properties and several lengthscale-specific physical features of bone, see Open Issue 2, can be quantified, or estimated, through medical imaging techniques, observation and experiments $10A_{1}$, $10A_{2}$.

There are three main reasons to perform multiscale fracture analysis on bone [377,378]:

$13A_{1}$: each lengthscale exhibits specific geometries, mechanical properties and physical features;

$13A_{2}$: each lengthscale is directly influenced by the geometry, mechanical properties and physical features of the nearest lower-scale, see Definition 9;

$13A_{3}$: fracture and several other physical phenomena start at the molecular scale.

**Remark** **28** (Scales Classification). *There is no “standard" classification for devising bone lengthscales. The surveyed literature sometimes refers to the same lengthscale geometry or physical feature at different lengthscales. For example, unlike [139], ref. [378] does not define a mesoscale. Furthermore, what [378] illustrates as the sub-nanoscale, ref. [139] illustrates as the nanoscale of bone. Thus, when modelling different bone lengthscales, it is important to define the geometric features and characteristic length of each lengthscale.*

**Figure 4:**
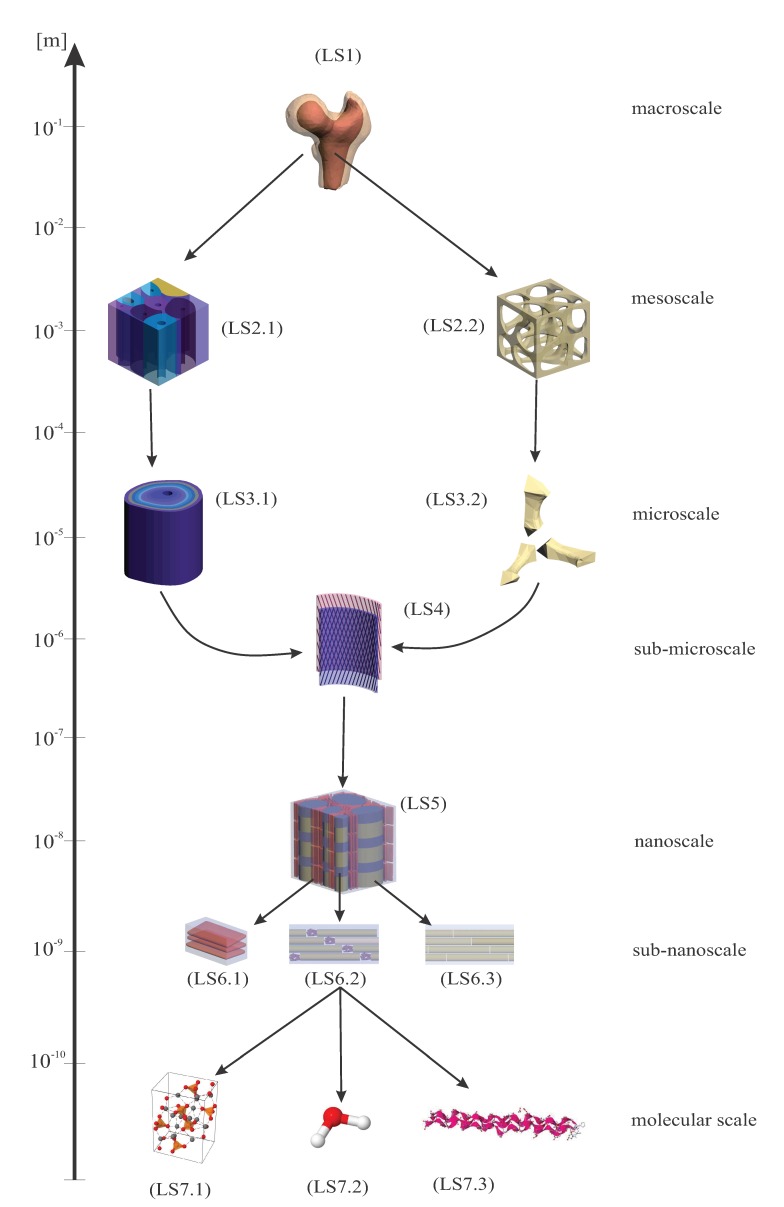
The multiscale structure of bone.

Figure 4 illustrates the bone lengthscales found in the reviewed literature. Each lengthscale is discussed in the following subsections.

The continuum mechanics approach shown in Figure 1 can be applied to any lengthscale that is coarse enough to be modelled as a continuum. The molecular scale should best be assumed as a non-continuum.

### 13.1. Molecular Scale—$H_{2}O$-CLG-HA Lengthscale

At the molecular scale, see Figure 4, bone is composed of three components: water *LS7.2*, an organic phase *LS7.3* and an inorganic phase *LS7.1*. Water ($H_{2}O$) represents approximately 10% of total bone mass. The organic phase represents 30% of total bone mass and is 90% constituted by of type I ColLaGen (CLG) and 10% by of a combination of other collagen types (III and VI) plus non-collagenous proteins. The inorganic phase of bone is a ceramic crystalline-type mineral labelled hydroxyapatite (HA): $Ca_{10}{\left(PO_{4}\right)}_{6}{\left(OH\right)}_{2}$ [61,379] and represents 60% of total bone mass [380].

**Characteristic Length 1** (Molecular Scale).

HA mineral crystal and CLG molecule: HA length 20–200 nm [61,139,191,378,379,381,382,383] HA width 15–70 nm [61,139,191,378,379,382,383] HA thickness 1.5–5 nm [61,139,191,378,379,382,383] CLG diameter 1.5–3.5 nm [61,139,378] CLG length 300 nm [61,139,222,378,384]

**Remark** **29** (Proportion of HA, CLG and $H_{2}O$ Contents). *Bone’s HA, CLG and $H_{2}O$ contents vary from species to species, from individual to individual and from one anatomical location to another. Yet, the average chemical composition of healthy bone inside a large-enough cube-shaped volume remains constant in space (i.e., across all cube-shaped volumes comprising the whole bone) and in time (i.e., along the aging process, starting from early adulthood) [115,140]. Since bone CLE-properties depend mostly on the proportion between its HA, CLG and $H_{2}O$ contents, different proportions translate into correspondingly different CLE-properties [115,140,286,287,288,289,290,291,385].*

Collagen alone displays a multiscale structure [386,387,388]. A single collagen molecule, a tropocollagen, is a helical structure consisting of three left-handed polypeptide chains coiled around each other to form a right-handed superhelix. What is known as collagen is actually tropocollagen, the basic triple-helical structural unit of collagen, i.e., a single collagen molecule.

It is in the molecular lengthscale where vitamin deficiency, sunlight exposure time, physical activities and other variables presented in Open Issue 2 are hidden. They alter, among others, the distribution and arrangement of atoms and molecules. Changes in the physical structure and chemical composition at the molecular lengthscale imply changes in geometry and mechanical properties at higher-scales, see Definition 9. These changes have a direct influence on osteoporosis and bone fracture.

### 13.2. Sub-Nanoscale—Mineralized Collagen Fibrils Lengthscale

At the sub-nanoscale, a collection of axially connected CLG molecules located next to each other in a thread-like structure with a high length-to-diameter ratio forms a collagen fibril. The fibrillar structure exhibits an axial periodicity, with gaps between the end of the collagen molecules of ∼40 nm [383]. Zones across the length of a fibril with gaps are labelled gap zones, see Figure 4 *LS6.2*. Zones across the length of a fibril with no gaps are labelled overlap zones, see Figure 4 *LS6.3*. Gap and overlap zones appear periodically with a characteristic distance (D) of ∼67 nm [383,389,390]. Deposition of the HA crystals, see Figure 4 *LS6.1*, occurs within gap zones [384]. A CLG fibril with HA deposition is labelled mineralized CoLlaGen fibril (mCLGf), see Figure 4 *LS6.2*.

HA crystals are also found surrounding and oriented parallel to mCLGfs, i.e., in the extra-fibrillar volume. The extra-fibrillar volume is a foamlike structure basically composed of HA crystals and filled with $H_{2}O$ and a small portion of non-collagenous organic matter [61,391]. The major portion of HA crystals, in bone, is located in the extra-fibrillar volume. HA crystals located in the extra-fibrillar volume are larger than HA crystals located within the gap zones of mCLGf. Several experimental approaches confirm the existence of HA in the extra-fibrillar volume, e.g., neutron diffraction [392,393] and electron microscopy [380,382,383,391,394,395,396]. Furthermore, many works have modelled bone considering HA to be also outside CLG fibrils [142,385,397,398,399].

**Remark** **30** (Mineral Within Bone). *As well explained in [380], there are two views of the sub-nanoscale of bone: an older—and nowadays less accepted—view, which considers HA to be located only within the gap zones of collagen fibrils, building mCLGfs; and a more recent—and more consistent—view, which considers HA to be mostly located in the extra-fibrillar volume, outside mCLGfs.*

**Characteristic Length 2** (Sub-nanoscale).

Mineralized Collagen Fibril: mCLGf diameter 20–150 nm [61,139,378,379,400,401,402] mCLGf length 10,000–30,000 nm [401,403,404] CLGs gaps 35–44 nm [61,139,222,378,379]

**Highlight** **9.** *The influence of mechanical properties of the mCLGf and of the extra-fibrillar volume (or matrix) on the mechanical properties of trabecular bone was recently investigated by [405]. The extent of modifications in energy to failure during mCLGf rupture and separation relative to the changes in the properties of the mCLGf and the extra-fibrillar volume was quantified.*

### 13.3. Nanoscale—Collagen Fiber Lengthscale

At the nanoscale, arranged bundles of collagen fibrils, separated from each other by a thin layer of extra-fibrillar volume, form collagen fibers [206,222,381], see Figure 4 *LS5*.

**Characteristic Length 3** (Nanoscale).

Collagen Fibers: CLG fiber diameter 0.15–0.25/2–3 µm [378]/[402,406] CLG fiber length ≈10–30 µm several mCLGf lengths

**Remark** **31** (Fibers and Fibrils). *Some of the surveyed literature seems to interchange the words fiber and fibril. Both are thread-like structures with a high length-to-diameter ratio, but fibers are larger and thicker than fibrils. A bundle of fibrils characterizes a fiber [222].*

### 13.4. Sub-Microscale—Lamella Lengthscale

A group of collagen fiber layers, each layer containing an arrangement of unidirectional fibers, is labelled lamella, see Figure 4 *LS4*.

**Characteristic Length 4** (Sub-microscale).

>Lamella: lamella length ≈10–30 µm several CLG fiber lengths lamella width ≈0.15–0.25 µm several CLG fiber diameters lamella thickness 3–7 µm [61,139,191,379]

### 13.5. Microscale—Osteon and Trabecula Lengthscale

Different assemblies of lamellae give origin to two different types of bone: cortical and trabecular. The cortical bone consists of osteons and Haversian canals, see Figure 4 *LS3.1*. The trabecular bone consists of many single trabeculae, rod-like structures, arranged in a porous way, see Figure 4 *LS3.2*. Osteons and trabecula are the lengthscale elements that define the microstructure of cortical and trabecular bone, respectively.

**Characteristic Length 5** (Microscale).

Osteon and Trabecula: osteon length 10,000–20,000/1000–3000 µm [222]/[379] osteon diameter 200–300 µm [191,222,379] trabecula length 1000 µm [191] trabecula thickness 50–300 µm [61,139,191,379]

**Remark** **32** (Bone Porosities). *At the microscale, bone is composed of a solid structure with porosities. The solid structure, composed of lamellae, is commonly labelled solid bone matrix or bone ultrastructure and may be considered tissue-independent [385]. Cortical bone exhibits pores as Haversian and Volkmann’s canals. Trabecular bone exhibits several pores in the intertrabecular spaces. Bone pores are filled either with a fluid or a gel, e.g., blood vessels, nerves, fat, bone marrow, et cetera [222].*

### 13.6. Mesoscale—Cortical and Trabecular Bone Lengthscale

The cortical bone, see Figure 4 *LS2.1*, also labelled compact bone, and the trabecular bone, see Figure 4 *LS2.2*, also labelled cancellous or spongy bone, constitute the lengthscale that lays between the micro- and macroscales. This scale is commonly labelled mesoscale in the literature of bone multiscale modelling.

**Remark** **33** (Mesoscale—Multiscale Literature). *The multiscale modelling literature defines mesoscale as any intermediate lengthscale, i.e., any lengthscale that is not the finest or the coarsest modelled lengthscale [407] (pp. 6, 214). For example, in the case of three lengthscales consideration, the mesoscale is defined as a scale with a characteristic length $L_{\mathrm{meso}}|L_{\mathrm{micro}}<L_{\mathrm{meso}}\ll L_{\mathrm{macro}}$, where $L_{\mathrm{micro}}$ and $L_{\mathrm{macro}}$ are the characteristic length of the micro- and macroscales, respectively [385,408,409], see Definition 8.*

**Characteristic Length 6** (Mesoscale—Representative Volume Element (RVE)). *Defined in the Multiscale literature as a scale between any two lengthscales, see Remark 33, bone mesoscale is usually referred to as the scale between the macro- and microscale in the literature regarding bone modelling. The mesoscale is characterized by an RVE, usually cube-shaped, that contains several elements of the microscale, e.g., a bunch of osteons or a bunch of trabeculae, see Figure 4 LS2.1 and LS2.2. An appropriate characteristic length for the mesoscale of bone is 10 to 100 times the characteristic length of the microscale.*

### 13.7. Macroscale—Whole Bone Lengthscale

At the macroscale, the whole bone, consisting of cortical and trabecular bone, is considered, see Figure 4 *LS1*.

**Characteristic Length 7** (Macroscale—Whole Bone). *Different bones have different sizes. The femur is the longest human bone. The mean ratio of femur length to human stature is approximately 26.74% [410]. For instance, a 1.7 m tall person has a 45 cm long femur. The stapes is the smallest bone in humans. The distance between the surface of the head of the stapes to the surface of its footplate, i.e., its greater length, is approximately 3.19 mm [411].*

At the macroscale, bone can be classified based on its skeletal site, shape and structure [412]. According to its structure, bone can be classified into cortical and trabecular bone, see Figure 4 *LS2.1*, *LS2.2*. According to their shape, bones can be classified into five different groups: (**1.**) long bones; (**2.**) short bones; (**3.**) flat bones; (**4.**) irregular bones; (**5.**) sesamoid bones. The huge majority literature on bone fracture simulation focuses on long bones, probably due to its beam-like geometry that enables simplified analytical calculations and experimental reproducibility.

Other types of tissue found in bone, but not discussed in detail in this paper, include bone marrow, endosteum, periosteum, nerves, blood vessels and cartilage. They also play a role in bone fracture.

References [61,96,139,180,191,206,222,378,379,381,384,385,386,400,402,412,413,414,415,416,417] constitute the main literature concerning the elaboration of Section 13 and are recommended for further details.

## 14. Multiscale Modelling of Bone

Multiscale modelling of bone starts when bone is assumed to comprise at least two of the lengthscales presented at Section 13, and consists of linking at least two different lengthscales. Each scale is distinguished by its characteristic length, see Definition 8.

**Definition** **8** (Characteristic Length). *The characteristic length of a lengthscale quantitatively describes the physical space occupied by the RVE of this lengthscale. An RVE must contain enough physical space to enclose a fully defined example of all physical phenomena that were assumed to take place at a certain lengthscale. For example, in the case of cubic-shaped RVEs, the edge length of the RVE is, in most cases, the most suitable choice of characteristic length.*

The linking between lengthscales can either be done by transitioning from a certain scale $LS_{x}$ to a *lower-scale* (Downscaling) $LS_{x+1}$ or from a certain scale $LS_{x}$ to a *higher-scale* (Upscaling) $LS_{x-1}$, see Definition 9.

**Definition** **9** (Higher- and Lower-Scales). *Picture two lengthscales. The higher lengthscale, or higher scale, is the lengthscale with greater characteristic length. The lower lengthscale, or lower-scale, is the lengthscale with shorter characteristic length.*

At a certain lengthcale $LS_{x}$, the domain of a solid body can either be described as a continuum or as a conjunction of discrete particles, i.e., non-continuum. Two distinct lengthscales may have one of three possible relationships outlined in [418]: hierarchical, semi-concurrent and concurrent; further discussion of these three relationships is outside the scope of this survey. All 3 relationships may characterize continuum–continuum scale transitions or continuum–discrete scale transitions.

Table 3 shows the bone lengthscales modelled by the surveyed papers. Papers which performed multiscale analysis are present in more than one box in Table 3. There are difficulties in applying hierarchical multiscale models to fracture [418].

### 14.1. Continuum Downscaling

In a solid continuum, the transition from a certain lengthscale $LS_{x}$ to a lower-scale $LS_{x+1}$ is commonly labelled Downscaling or Localization [407]. Details on localization techniques are presented in [418,421]. Within the surveyed literature, continuum downscaling consists in transferring the displacement and surface force vector fields or stress and strain tensor fields calculated at points inside the spatial domain of $LS_{x}$ as suitable BCs to the boundaries of the RVEs of $LS_{x+1}$ [422,423,424,425,426].

There are three main classes of BCs used for downscaling: **Periodic Boundary Conditions** (PBCs) are the most used BCs for spatial downscaling. As an advantage, they provide the fastest convergence of physical and mechanical properties of $LS_{x}$. As a disadvantage, the fact that they restrict the deformation to obey the structural frame periodicity of $LS_{x+1}$ imposes unphysical deformation constraints over localization zones (i.e., regions of relative extremely high deformation gradient where micro-cracks occur) [425,426]. **Minimal Kinematic Boundary Conditions** (MKBCs) ensure effective deformation shear strain but overestimate the number of localization zones near the domain boundary [425]. **Tesselation Boundary Conditions** (TBCs) maintain the point-to-point conditions imposed by PBCs while shifting the periodicity frame to correspond to the developing localization zone. In biomaterials, e.g., bones, when transitioning from $LS_{x}$ to $LS_{x+1}$, TBCs may give the least-error estimation of stresses and strains at $LS_{x}$ [426]. Four references feature bone multiscale analyses with downscaling: [53,69,83,143], see Table 3. To transition from the macroscale to the mesoscale, ref. [69] used a displacement interpolation procedure. To transition from the mesoscale to the microscale, ref. [69] transferred mesoscale displacements as BCs to the microscale. Likewise, ref. [143] transitioned from the macroscale to the mesoscale by applying displacements computed from the macroscale strain tensors as BCs on the boundary of the mesoscale RVE.

To transition from the macroscale to the microscales, and vice versa, [53,83] used intermediate scales, i.e., mesoscales. This transition was performed using octree hierarchical multiresolution geometric data structure. This kind of transition consists in mesh refinement. There is little controversy regarding the assumption of mesh refinement as a multiscale approach.

### 14.2. Continuum Upscaling

In a solid continuum, the transition from a certain lengthscale $LS_{x}$ to a higher-scale $LS_{x-1}$ is commonly labelled Upscaling or Homogenization [407]. Details on homogenization techniques are presented in [418,421,427]. Within the surveyed literature, continuum upscaling consists of averaging the displacement and surface force vector fields or stress and strain tensor fields calculated in points inside the spatial domain of $LS_{x}$ to displacement and traction vector fields at $LS_{x-1}$ by using averaging-based homogenization techniques [423,424].

Consider a stress tensor $T^{LS_{x}-1}$ and a strain tensor $E^{LS_{x-1}}$ at $LS_{x-1}$, and a point x inside an RVE’s domain $\Omega_{LS_{x}}$ at $LS_{x}$, i.e., $x\in \Omega_{LS_{x}}$. Homogenization evaluates, at any time instant *t*, $T^{LS_{x-1}}$ and $E^{LS_{x-1}}$ as the volume average of $T^{LS_{x}}$ and $E^{LS_{x}}$ over $\Omega_{LS_{x}}$ [422,424]:

(4) $$\begin{array}{c} T_{ij}^{LS_{x-1}}\left(x\left(t\right),t\right)=\langle T_{ij}^{LS_{x}}\left(x\left(t\right),t\right)\rangle =\frac{1}{|\Omega_{LS_{x}}|}\int_{\Omega_{LS_{x}}}^{}T_{ij}^{LS_{x}}\left(x\left(t\right),t\right)d\Omega_{LS_{x}}\\ E_{ij}^{LS_{x-1}}\left(x\left(t\right),t\right)=\langle E_{ij}^{LS_{x}}\left(x\left(t\right),t\right)\rangle =\frac{1}{|\Omega_{LS_{x}}|}\int_{\Omega_{LS_{x}}}^{}E_{ij}^{LS_{x}}\left(x\left(t\right),t\right)d\Omega_{LS_{x}} \end{array}$$

where $|\Omega_{LS_{x}}|$ is the volume of $\Omega_{LS_{x}}$ in absolute value. All of the surveyed literature concerning upscaling procedures in bone used Equation (4), see Table 3 rows **c** and **d**. For example, ref. [140,141] present a cascade homogenization procedure for transitioning between several lengthscales. When transitioning from any $LS_{x}$ to any corresponding $LS_{x-1}$, Equation (4) interprets any spatial discontinuity within the RVE of $LS_{x}$ as a uniform volumetric redistribution of $T^{LS_{x}}$ and $E^{LS_{x}}$ over all space enclosed by the RVE.

#### Molecular Scale as a Non–Continuum Material

At the molecular scale, bone is composed of a colossal number of interacting molecules, see Section 13. Each molecule comprises several atoms participating in interatomic bonds. Assuming that modelling each atom as a solid particle is accurate enough, the molecular domain is defined, in conclusion, as a gathering of discrete particles, i.e., a non-continuum. The molecular scale of bone is mostly studied through Molecular Dynamics (MD) simulations [386,428,429].

**Remark** **34** (Linking Continuum and Non-Continuum Scales). *None of the surveyed literature transitions, in bone, between a lengthscale modelled as a continuum and a lengthscale modelled as a non-continuum. Within the surveyed literature, only [140,290,291] model the molecular scale alongside larger lengthscales of bone, see Table 3; however, ref. [140,290,291] model the molecular scale as a continuum using a multiscale micromechanics-based approach. This approach can estimate bone anisotropic CLE-properties from $H_{2}O$, HA and CLG, the basic constituents of bone, see Section 13, and may be combined with CT-data to estimate patient-specific CLE-properties [61,115,140,286,287,288,289,290,291].*

The transition between a lengthscale modelled as a non-continuum and a lengthscale modelled as a continuum can be performed using several approaches; ref. [427] further comments on these possible approaches. This literature survey emphasizes an approach consisting in finding parameters for Traction-Separation Equations (TSEs) through MD simulations, as outlined in [418,430,431]. TSEs describe fracture at any lengthscale that is modelled as a continuum material. The TSE parameters are used by CZMs that describe fracture as element-wise interface disconnection; lastly, the CZM is coupled with the Governing Equations (1)–(3) [430,432,433,434,435].

MD simulations, as proposed by [436], and performed in bone by [191,414,415,419,420,437,438], consist in solving Newton’s 2nd Law of Motion at a material’s molecular scale whose spatial domain contains *a* atoms interacting with up to *n* neighbour atoms:

(5) $$\;m_{a}\frac{d^{2}r_{a}\left(t\right)}{dt^{2}}=\sum_{n_{1}=1}^{n}{𝕗}_{2}\left(r_{a}\left(t\right),r_{n_{1}}\left(t\right)\right)+\cdots +\sum_{n_{1}=1}^{n}\sum_{\begin{array}{c} n_{2}=1\\ n_{2}\neq n_{1} \end{array}}^{n}\cdots \;\sum_{\begin{array}{c} n_{k}=1\\ n_{k}\neq n_{1},n_{2}\ldots \end{array}}^{n}\;{𝕗}_{n}\left(r_{a}\left(t\right),r_{n_{1}}\left(t\right),\cdots ,r_{n_{k}}\left(t\right)\right)$$

where, for each *a*-th atom: $r_{a}$ is the position vector; $m_{a}$ is the mass, ${𝕗}_{2}$ is a force vector function that describes pairwise atomic interactions; similarly, ${𝕗}_{n}$ describes *n*-atom interactions. Each ${𝕗}_{n}$ is the time-derivative of an energy function that accounts for up to *n*-body and quantum interactions. The total energy of the *a*-th atom is a function of an *a*-th atom’s position $r_{a}\left(t\right)$ and of its *n* neighbours’ positions $r_{1}\left(t\right),\cdots ,r_{n}\left(t\right)\in R^{3}$.

MD simulations of bone commonly account for the presence of CLG, HA and H${}_{2}$O, see Section 13 and [191,386,414,415,419,420,428,429,437,438]. For each **inter**- and **intra**-molecular interaction there are specific potential energy functions (or simply potentials), some of which are found in the literature referred to in Table 4.

When formulating energy functions for the CLG–HA, CLG–$H_{2}O$ and HA–$H_{2}O$ inter-molecular interactions, the Lorentz-Berthelot mixing rule [440,441] is usually used, as in [414,415].

Once ${𝕗}_{2},\cdots ,{𝕗}_{n}$ are defined, the next step of an MD simulation consists either in solving: (**1.**) an initial-value problem, which requires the atoms’ initial positions r(0) and velocities $\dot{r}\left(0\right)$; or (**2.**) a boundary-value problem, which requires the atoms’ positions at an initial time instant $r(t_{i})$ and at a final time instant $r(t_{f})$. Both (**1.**) and (**2.**) require potential energy functions.

MD simulations provide the position $r_{a}\left(t\right)$ and velocity ${\dot{r}}_{a}\left(t\right)$ of every *a*-th atom in the molecular spatial domain at each time step. The accuracy of MD simulation results depends strongly on the inter- and intra-molecular potential’s validity. Therefore, the before mentioned 6 inter- and intra-molecular potentials must be selected according to their accuracy in modelling bone mechanics at the molecular scale [413,414].

**Highlight** **10** (Multiscale Modelling and Bone Remodelling). *Bone remodelling, see Definition 19, has been recently incorporated into multiscale models. The first approach to study bone remodelling by coupling models of systems biology and multiscale continuum micromechanics is presented in [445]. Folllow-up papers studied the relationship between oscillating hydrostatic pore pressure and bone cells activity [446,447], which is presented from a biological perspective in [448].*

*A comprehensive multiscale model of bone remodelling multiscale model, accounting for hormonal regulation and biochemical coupling of bone cell populations is presented by [245]. Structural changes induced by osteoclasts and osteoblasts at the “cellscale” change bone density at higher-scales in the model proposed by [449].*

**Open Issue** **9** (Simulations Coupling 6 or More Lengthscales). *No paper has been found modelling and linking all the lengthscales illustrated in Figure 4 and Table 3. Among the surveyed literature, ref. [290,291] devised the multiscale model with the largest number of linked lengthscales: 5.*

**Remark** **35** (Scales Jumps). *Table 3 row* *d* *shows scale jumps performed by [138,290,291]. Jumping from the sub-nanoscale directly to the sub-microscale, as in [138], may be justified by the fact that mCLGf and CLG fibers exhibit the same geometry. CLG fibers are nothing but a bunch of mCLGfs. Thus, it may be reasonable to skip the transition between the sub-nanoscale and the nanoscale of bone. Jumping from the sub-microscale directly to the mesoscale, as in [290,291], may be justified by the fact that the microscale is composed of, in the case of cortical bone, a single osteon and the mesoscale is a bunch of osteons, see Characteristic Length 6.*

## 15. Validating Bone Fracture Simulation

Simulations should imitate reality as accurately as possible. The way to validate solid continuum mechanics models is by performing simulations that imitate real-world experiments. A model is labeled validated when the numerical value of a variable computed in a simulation satisfactorily matches the numerical value from the experimental measurement of the same variable. For instance, the numerical value of a strain computed from a simulation must match the numerical value of a strain measured in an experiment representative of said simulation.

There are mainly two classes of experiments on bone material: $15A_{1}$ and $15A_{2}$. Both involve highly sensitive procedures that must be carefully performed, see guidelines on biomechanical experiments by [233].

$15A_{1}$: Experiments for Bone Material Characterization—give insight into the mechanical behaviour of bone under specific loading conditions and into how Open Issue 2 affects bone mechanical properties. $15A_{1}$ are related to the bone modelling categorization shown in Table 2 and described in Section 10: $10B_{1}$–$10B_{3}$. $15A_{1}$ must comprehend the largest possible number of variables, see Remark 3. $15A_{1}$ must test the specimen: at all possible lengthscales, see Section 13; at all possible loading conditions, e.g., tension, compression, shear, torsion; at all directions, i.e., assuming every planes of symmetry, e.g., assuming the material to be triclinic; under different strain rates; under different thermal conditions, e.g., different temperatures, temperature change rates. Furthermore, $15A_{1}$ must characterize: the plastic region; fracture toughness mechanisms and other important features [221,326,450,451,452]. $10B_{1}$–$10B_{3}$ that best suit experimental results must be sought after.

$15A_{2}$: Experiments on Bone Materials under Generic Environmental Conditions—must imitate in vivo, see Definition 1, conditions (when possible) and consider several issues to assure reproducibility, e.g., bone conservation, hydration, temperature, et cetera.

**Remark** **36** (Validation and Experiments—An Iterative Process). *Mechanical properties of bone must be modelled based on preliminary $15A_{1}$ that estimate $10B_{1}$–$10B_{3}$. Simulated variables, e.g., strains, displacements, et cetera, are validated by being compared to $15A_{2}$. When simulated variables do not match $15A_{2}$ (assuming both experiments and simulations were correctly performed), a new model, with different assumptions, based on new $15A_{1}$, is required. This is an iterative process. $15A_{1}$ are required pre-simulation. $15A_{2}$ are required post-simulation.*

**Remark** **37** (Validated?). *Some references in Table 2 made simple assumptions, e.g., [30,81,115,127] modelled bone only at the macroscale and/or as an isotropic homogeneous CLE-material, and validated their simulations, i.e., their simulation showed good agreement with $15A_{2}$. It is reasonable to question such results: bone is most probably not isotropic and does not fit an LE constitutive equation, see Section 10. $15A_{1}$ may even reveal some particular bone sample to behave like a quasi-isotropic homogeneous CLE-material; however, these findings may stop being valid under the slightest change in local conditions, e.g., bones in vivo vs. bones in vitro, et cetera.*

Experiments and simulations contain errors and approximations; thus, they are always questionable.

**Remark** **38** (Towards Realistic Fracture Predictions). *Most realistic fracture predictions stem from the best-achievable fracture simulations. To achieve such simulations the following issues should be considered: (* ***1.*** *) the multiscale structure of bone, see Section 13 and Section 14; (* ***2.*** *) the most robust physico-mathematical approach, see Section 6, Section 7 and Section 8 and Section 12; (* ***3.*** *) the most realistic mechanical properties, see Section 10; (* ***4.*** *) the most realistic BCs, see Section 11; (* ***5.*** *) proper validation, see Section 15.*

## 16. Assessing Fracture Risk

Current available osteoporosis and fracture risk assessment tools are fundamentally based on BMD and qualitative medical variables (e.g., sex, age, weight, patient’s case history), most commonly labelled Clinical Risk Factors (CRFs) [453].

There are single-variable and multi-variable fracture risk predictors and assessment tools.

### 16.1. Single-Variable Risk Analysis

**BMD** measurements are the current clinical standard to diagnosis osteoporosis. According to the WHO, women with a BMD that lies 2.5 SD or more below the average value for young healthy women are classified as *osteoporotic* (T-score ≤2.5 SD) [11]. However, BMD alone is unable to accurately assesses fracture risk [454]. Patients classified as osteoporotic will not invariably suffer a fragility fracture; non-osteoporotic patients may also suffer a fragility fracture [11,14,455]. BMD can be used in conjunction with CRFs and available fracture assessment tools to improve the accuracy of fracture predictions. CRFs provide information on fracture risk that are unrelated to BMD [165]. As mentioned in Section 9, DXA is the clinical standard technique to measure BMD. QCT and ultrasound measurements are alternative techniques for the quantification of BMD [14,153].

**TBS**, an acronym for Trabecular Bone Score, is a grayscale-based (or HU-based) texture measurement influenced by the geometry of bone at the meso- and microscales [456]. A low TBS value may indicate thin trabeculae and a highly porous mesostructure, see Figure 4 (LS2.2, LS3.2) [457]. TBS contains structural information that are not captured by BMD measurements [457,458]. TBS can be used, though not very accurately, to assess fracture risk independently of BMD and CRFs [453,459]. TBS has been used in conjunction with BMD alone and with BMD and CRFs in available fracture risk assessment tools such as FRAX, to improve the accuracy of bone fracture predictions [458]. In the study performed by [459], however, TBS did not improve BMD and FRAX fracture predictions. TBS depends on HU variations obtained in vivo, which can have many causes [457] and is most commonly estimated using DXA. For more information on TBS see [14,459,460,461,462].

**BTMs**, an acronym for Bone Turnover Markers, are measurable indicators of bone turnover, e.g., blood and urine tests. Bone turnover, i.e., bone replacement, is the effect, the cause (mechanism) of which is bone remodelling, see Definition 19. Bone turnover refers to the volume of replaced bone per unit time [463]. Deterioration of bone microstructure, i.e., bone structure at the microscale, translates into a high value of bone turnover. BTMs indicate the degree of deterioration of the bone microstructure and, may thus, independently of BMD, predict a person’s fracture risk. Furthermore, BTMs can be used in conjunction with BMD to improve the accuracy of fracture risk assessment tools [464]. The use of BTMs in the osteoporotic risk analysis and in monitoring the efficacy of osteoporosis treatment is rapidly increasing [465,466,467,468].

### 16.2. Multi-Variable Risk Analysis

**FRAX** (https://www.sheffield.ac.uk/FRAX/), the Fracture Risk Assessment tool, estimates individualized ten-year probability of hip, spine, forearm and proximal humerus osteoporotic fracture [469,470]. FRAX integrates eight main CRFs (prior fragility fracture, parental hip fracture, smoking, systemic glucocorticoid use, excess alcohol intake, body mass index, rheumatoid arthritis, and other causes of secondary osteoporosis), which, in addition to age and sex, contribute to fracture risk analysis independently of BMD. FRAX does not consider risk factors such as BTM and those associated with falls, lower dietary calcium intake and Vitamin D deficiency [464], but has BMD as an optional input variable [165,469,470]. FRAX predictions can become more accurate when used in conjunction with, e.g., BMD and TBS [458]. For more information on FRAX, see [41,91,453,471,472,473,474,475].

**QFracture** (https://qfracture.org/) predicts individual risk of osteoporotic and hip fracture based on several distinct CRFs (age, body mass index, ethnic origin, alcohol intake, smoking status, chronic obstructive pulmonary disease or asthma, any cancer, cardiovascular disease, dementia, diagnosis or treatment for epilepsy, history of falls, chronic liver disease, Parkinson’s disease, rheumatoid arthritis or systemic lupus erythematosus, chronic renal disease, type 1 and 2 diabetes, previous fracture, endocrine disorders, gastrointestinal malabsorption, any antidepressants, corticosteroids, unopposed hormone replacement therapy and parental history of osteoporosis), needing no quantitative measurements [476,477]. When compared with FRAX, QFracture shows some evidence of improved discrimination and calibration for hip fracture [476]. BMD cannot be used in conjunction with QFracture.

**Garvan**, short for Garvan Fracture Risk Calculator [478,479], estimates individualized five- to ten-years risk of total fracture and hip fracture by combining BMD and several CRFs (age, body weight, height, daily physical activity level, daily calcium intake, smoking, history of falls in the preceding 12 months, history of fractures in the past five years, et cetera [480]).

Comparisons between FRAX, Qfracture and Garvan discussing approaches to osteoporosis risk analysis worldwide can be found in [481,482,483,484]. The use of such risk assessment tools in conjunction with BMD, TBS, genetic data, BTMs and new CRFs could improve their accuracy in predicting fracture risk [481,485]. Furthermore, artificial intelligence could also enhance the accuracy of these tools, e.g., machine learning methods can be used to assess the risk of osteoporotic fractures [486].

Others fracture risk assessment tools are available in the literature: The DVO-Tool developed by the German Osteology Society [487,488], the fracture risk score model based on in-hospital treated patients to predict osteoporotic fractures [489], and the FRACTURE Index [490] are only a few of them. A brief schematic overview of the evolution of the diagnosis of osteoporosis since 1940 is given in [491].

Current available fracture risk assessment tools generally evaluate individual fracture risks based on a cohort of statistical data. However, current tools do not account for bone mechanical properties and/or bone quantitative fracture mechanics variables. Fracture simulation of patient-specific computational models could give already existing risk assessment tools its contribution by adding diagnostic quantitative information based on multiscale simulations.

**Open Issue** **10** (Quantitative Risk Factors). *Mathematical calculations can accurately assess the fracture risk of many different materials (e.g., steels, iron, composites) subject to specific BCs. It can possibly also accurately assess the fracture risk of biological materials. Continuum mechanics, fracture mechanics, multiscale modelling and molecular mechanics enable the designing of robust structures capable of supporting extreme (e.g., loading and thermal) conditions. Quantitative Risk Factors (QRFs), e.g., Young’s Modulus and Yield strength, could improve the accuracy of current fracture risk assessment tools.*

As shown in Section 13, bone presents different geometries and thus mechanical properties, see Section 10, at different lengthscales. The analysis of patient-specific fracture risk is a multiscale problem and requires consideration of as many bone lengthscales as possible [214]. Adding, from patient-specific bone computational models, quantitative multiscale fracture mechanics-based variables to current fracture risk assessment tools may be crucial to the improvement of current fracture risk analysis, being the next step towards an improved fracture-predictive diagnosis [128,214,481,485,492,493]. Not only osteoporosis, but several others bone diseases and conditions could profit from patient-specific fracture risk simulations and assessment tools, e.g.: osteomalacia, osteitis fibrosa, osteopenia (or bone loss), osteogenesis imperfecta, brittle bone disease, et cetera [194].

## Figures and Tables

**Table 1:** Main interested parties involved in bone fracture and which sections of this paper are most interesting for each party.

Specialization, Interested	Related Sections
**Biology**	Section 3. Motivating Patient-Specific Bone Fracture Simulation
	Section 4. Motivating this Literature Survey
	Section 6. The Physico-Mathematical Approach to Bone Fracture
	Section 7. Modelling Bone as a Continuum
	Section 8. Categorizing the Surveyed Literature into a Continuum Mechanics Framework
	Section 9. Patient-Specific Geometry of Bone
	Section 10. Mechanical Properties Categorization for Computational Bone Models
	Section 13. The Multiscale Structure of Bone
**Medicine**	Section 3. Motivating Patient-Specific Bone Fracture Simulation
	Section 4. Motivating this Literature Survey
	Section 6. The Physico-Mathematical Approach to Bone Fracture
	Section 7. Modelling Bone as a Continuum
	Section 8. Categorizing the Surveyed Literature into a Continuum Mechanics Framework
	Section 9. Patient-Specific Geometry of Bone
	Section 10. Mechanical Properties Categorization for Computational Bone Models
	Section 11. Mathematical Model of Bone Trauma-inducing Accident—The Boundary Conditions
	Section 15. Validating Bone Fracture Simulation
	Section 16. Assessing Fracture Risk
**Physics**	Section 3. Motivating Patient-Specific Bone Fracture Simulation
	Section 4. Motivating this Literature Survey
	Section 6. The Physico-Mathematical Approach to Bone Fracture
	Section 7. Modelling Bone as a Continuum
	Section 8. Categorizing the Surveyed Literature into a Continuum Mechanics Framework
	Section 9. Patient-Specific Geometry of Bone
	Section 10. Mechanical Properties Categorization for Computational Bone Models
	Section 11. Mathematical Model of Bone Trauma-inducing Accident—The Boundary Conditions
	Section 12. Simulating Bone Fracture
	Section 13. The Multiscale Structure of Bone
	Section 14. Multiscale Modelling of Bone
	Section 15. Validating Bone Fracture Simulation
	Section 16. Assessing Fracture Risk
**Engineering**	Section 3. Motivating Patient-Specific Bone Fracture Simulation
	Section 4. Motivating this Literature Survey
	Section 6. The Physico-Mathematical Approach to Bone Fracture
	Section 7. Modelling Bone as a Continuum
	Section 8. Categorizing the Surveyed Literature into a Continuum Mechanics Framework
	Section 9. Patient-Specific Geometry of Bone
	Section 10. Mechanical Properties Categorization for Computational Bone Models
	Section 11. Mathematical Model of Bone Trauma-inducing Accident—The Boundary Conditions
	Section 12. Simulating Bone Fracture
	Section 13. The Multiscale Structure of Bone
	Section 14. Multiscale Modelling of Bone
	Section 15. Validating Bone Fracture Simulation
	Section 16. Assessing Fracture Risk
**Government**	Section 3. Motivating Patient-Specific Bone Fracture Simulation
	Section 4. Motivating this Literature Survey
	Section 16. Assessing Fracture Risk
**Philanthropy**	Section 3. Motivating Patient-Specific Bone Fracture Simulation
	Section 4. Motivating this Literature Survey
	Section 16. Assessing Fracture Risk

**Table 2:**
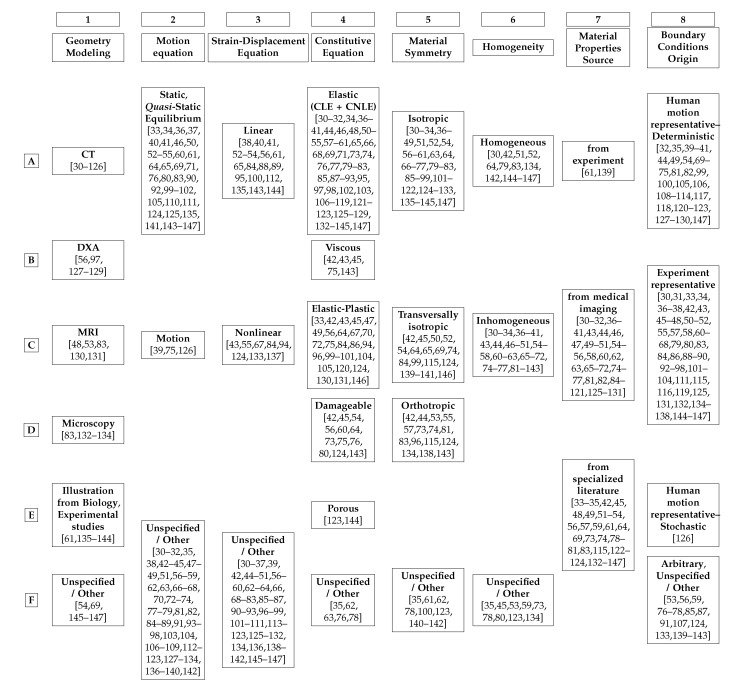
Categorization of surveyed literature that modelled bone as a solid continuum [30,31,32,33,34,35,36,37,38,39,40,41,42,43,44,45,46,47,48,49,50,51,52,53,54,55,56,57,58,59,60,61,62,63,64,65,66,67,68,69,70,71,72,73,74,75,76,77,78,79,80,81,82,83,84,85,86,87,88,89,90,91,92,93,94,95,96,97,98,99,100,101,102,103,104,105,106,107,108,109,110,111,112,113,114,115,116,117,118,119,120,121,122,123,124,125,126,127,128,129,130,131,132,133,134,135,136,137,138,139,140,141,142,143,144,145,146,147].

**Table 3:**
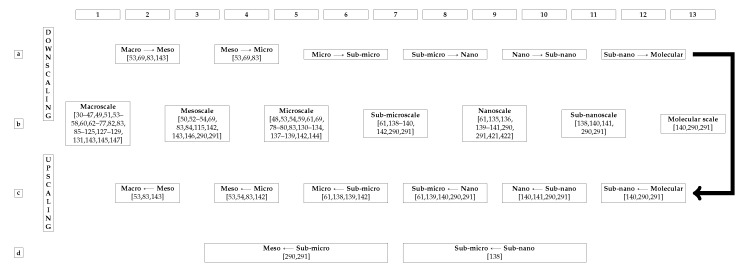
Bone multiscale modelling [30,31,32,33,34,35,36,37,38,39,40,41,42,43,44,45,46,47,48,49,50,51,52,53,54,55,56,57,58,59,60,61,62,63,64,65,66,67,68,69,70,71,72,73,74,75,76,77,78,79,80,82,83,84,85,86,87,88,89,90,91,92,93,94,95,96,97,98,99,100,101,102,103,104,105,106,107,108,109,110,111,112,113,114,115,116,117,118,119,120,121,122,123,124,125,127,128,129,130,131,132,133,134,135,136,137,138,139,140,141,142,143,144,145,146,147,290,419,420].

**Table 4:** Bibliographical references for interatomic potential function parameters.

Interaction	CLG–CLG	CLG–HA	CLG–$H_{2}O$	HA–HA	HA–$H_{2}O$	$H_{2}O$–$H_{2}O$
Reference	[413,415] *apud* [439]	[440,441]	[440,441]	[442] *apud* [437], [414] *apud* [443]	[440,441]	[413] *apud* [444]

## Appendix A. Glossary of Symbols

Symbol:	Meaning:
i,j,k,l=1,2,3	spatial components of Einstein’s summation or notation, or Index/Subscript/Tensor notion
$T_{ij}$	an element of the Cauchy stress tensor T $\left[N/m^{2}\right]\in R^{3x3}$
$b_{i}$	an element of the body force vector b $\left[N/m^{3}\right]\in R^{3x1}$
$f_{i}$	an element of the surface force vector f $\left[N\right]\in R^{3x1}$
$E_{ij}$	an element of the strain tensor E $\in R^{3x3}$
$n_{j}$	an element of the unit normal vector n $\in R^{3x1}$
ρ	a density $\left[g/m^{3}\right]\in R^{1}$
$X_{i}$	an element of the material point X $\left[m\right]\in R^{3x1}$ [494] (p. 61)
$x_{i}$	an element of the spatial point x $\left[m\right]\in R^{3x1}$ [494] (p. 61)
$F_{ij}$	an element of the deformation gradient F $\in R^{3x3}$
$u_{i}$	an element of the displacement vector u $\left[m\right]\in R^{3x1}$
$C_{ijkl}$	an element of the stiffness tensor C $\left[N/m^{2}\right]\in R^{3x3x3x3}$
*W*	the strain-energy density function $\left[J\right]\in R^{1}$
*t*	the time $\left[s\right]\in R^{1}$
θ	the temperature $\left[K\right]\in R^{1}$
$\Delta \theta_{i}$	an element of the temperature gradient Δθ $\left[K/s\right]\in R^{3x1}$
ξ	an unknown internal variable $\left[?\right]\in R^{?}$
*Y*	a unidirectional Young’s Modulus $\left[N/m^{2}\right]\in R^{1}$

## Appendix B. Glossary of Acronyms

Acronym:	Full form:
**BEM**	Boundary Element Method
**BC**	Boundary Condition
MK**BC**	Minimal Kinematic Boundary Condition
P**BC**	Periodic Boundary Condition
T**BC**	Tesselation Boundary Condition
**BMD**	Bone Mineral Density
**BTM**	Bone Turnover Marker
**CLG**	CoLlaGen
m**CLG**f	mineralized CoLlaGen fibril
**CT**	Computed Tomography
µ**CT**	Micro Computed Tomography
Q**CT**	Quantitative Computed Tomography
HR-pQ**CT**	High-Resolution Peripheral Quantitative Computed Tomography
**CZM**	Cohesive Zone Model
**DICOM**	Digital Imaging and Communications in Medicine
**DOF**	Degrees of Freedom
**DXA** or **DEXA**	Dual Energy X-ray Absorptiometry
**FDM**	Finite Difference Method
**FEM**	Finite Element Method
EP**FM**	Elastic-Plastic Fracture Mechanics
LE**FM**	Linear Elastic Fracture Mechanics
**FS**	Fall Stage
**GDP**	Gross Domestic Product
**HA**	HydroxyApatite
**HU**	Hounsfield Units
**LE**	Linear-Elastic
C**LE**	Cauchy-Linear-Elastic
CN**LE**	Cauchy-NonLinear-Elastic
N**LE**	Non-Linear-Elastic
**LS**	Lengthscale
**MD**	Molecular Dynamics
**MRI**	Magnetic Resonance Imaging
µ**MRI**	Micro Magnetic Resonance Imaging
**PDE**	Partial Differential Equation
**RVE**	Representative Volume Element
C**RF**	Clinical Risk Factor
Q**RF**	Quantitative Risk Factor
**SD**	Standard Deviations
**TBS**	Trabecular Bone Score
**TSE**	Traction Separation Equation
**WHO**	World Health Organization

## Appendix C. Glossary of PDE, Continuum Mechanics and Theory of Elasticity Concepts

References to several concepts important to the understanding of PDEs, continuum mechanics and theory of elasticity are listed here. These concepts have mathematical and physical enunciations. The mathematical enunciations are rigorous and contain all the details that computer simulations require. The physical enunciations give an intuitive idea, but are not mathematically rigorous and, in most cases, do not contain the necessary data required by the algorithm of a computer simulation. However, the physical interpretations facilitate interaction between biologists, physicians, engineers and physicists. Biologists and physicians need to understand definitions in physical terms to make suggestions to engineers and physicists so they can create more realistic bone models.

Some of the references listed on the physical definitions may also contain a mathematical definition. They are classified as a physical definition because they explain the concept in a more intuitive way. The references for the physical definition present the mathematical definition usually in a more easy-to-grasp way, sometimes not as complete and rigorous as the references for the mathematical definition. Furthermore, terms with multiple references may present slightly different approaches or interpretations in each reference.

Term:	Mathematical definition:	Physical definition:
Domain	([495] p. 222, same as *Gebiet*), [496] (p. 1, same as *Region*)	see Illustration 1
Boundary	[497] (pp. 25, 28, same as *Frontier*)	see Illustration 1
Boundary Condition	[498] (p. 23)	see Illustration 2
Constitutive equation	[499] (p. 170), [219] (p. 69), [500] (p. 1644), [494] (p. 276)	[222] (p. 170), [499] (p. 169), [206] (p. 35), [219] (p. 69), [501] (p. 273), [502] (p. 2), [494] (p. 223), [500] (p. 1642)
Elastic material (or Cauchy-elastic material)	[503] (p. 170), [504] (p. 207) [499] (p. 175), [502] (p. 117), [494] (p. 297)	[505] (p. 201), [506] (pp. 1, 444), [28] (p. 147)
Hyperelastic material (or Green-elastic)	[219] (p. 520), [503] (p. 171) [499] (p. 206), [502] (p. 294), [506] (p. 444)	[219] (p. 519), [502] (p. 13), [499] (p. 206), [28] (p. 148), [501] (p. 282)
Plastic (or elasto-plastic) material	[219] (p. 148), [506] (p. 131), [210] (p. 57)	[219] (p. 1480), [506] (p. 131), [210] (p. 52), [507] (p. 75)
Viscoelastic material	[503] (p. 174), [211] (p. 5)	[211] (p. 5), [222] (pp. 59, 217), [210] (p. 65)
Viscoplastic (or elasto-viscoplastic) material	[219] (p. 450)	[506] (p. 133), [210] (p. 65), [219] (p. 435)
Poroelasticity	[222] (p. 249)	[222] (p. 247)
Poroplasticity	[508] (p. 226)	[508] (p. 225)
Poroviscoelasticity	[508] (p. 261)	[508] (p. 261)
Poroviscoplasticity	[508] (p. 273)	[508] (p. 272)
Damage mechanics	[509] (p. 8) [510] (pp. 16, 142)	[511] (p. 1) [510] (p. 3) [512] (p. 1)
Isotropic material	[503] (p. 234), [502] (p. 78), [513] (p. 60), [504] (p. 243)	[514] (p. 25), [250] (p. 41), [505] (p. 203), [499] (p. 170)
Anisotropic material	[503] (p. 234), [502] (p. 78), [513] (p. 60), [504] (p. 243)	[514] (p. 25), [250] (p. 41), [505] (p. 203)
Linear elastic anisotropy: triclinic, monoclinic, orthotropic (or rhomibc), trigonal, tetragonal, transversally isotropic (or hexagonal), cubic, isotropic	[250] (p. 44), [222] (p. 150), [257] (p. 10)	[250] (p. 44), [222] (p. 150), [257] (p. 10)
Homogeneous material	[504] (p. 237), [502] (p. 58,59)	[502] (pp. 58, 59), [514] (p. 25), [505] (p. 203)
Inhomogeneous (or non-homogeneous, heterogeneous) material	[504] (p. 237)	[514] (p. 25), [505] (p. 203)
Properties (not only mechanical properties): global and local	[515] (p. 83)	[515] (p. 83), [516] (p. 532)
Cauchy’s equation of motion (equilibrium equation)	[504] (p. 223), [503] (pp. 153, 204), [499] (p. 148), [494] (pp. 139, 273, 307)	[222] (p. 129), [222] (p. 196)
Strain-displacement equation (or Lagrange strain tensor, strain)	[503] (p. 272), [505] (p. 84)	[206] (p. 29), [505] (p. 84)
Stress (or Cauchy stress tensor)	[504] (p. 174), [503] (p. 150) [494] (p. 137)	[206] (p. 25), [222] (p. 122), [505] (p. 157)
Displacement (or displacement vector)	[503] (p. 272), [494] (p. 297)	[206] (p. 30), [505] (p. 81)
Body force	[504] (p. 151), [503] (p. 97), [494] (p. 132)	[499] (p. 144), [494] (p. 132)
Surface force (or surface traction, stress vector, Cauchy traction field)	[503] (p. 97), [494] (p. 133)	[494] (p. 133), [206] (p. 26), [505] (p. 155)
Materials with memory	[504] (p. 201)	[502, XVIII preface to third edition]
Hookean Material (or generalized Hooke’s law, Hooke’s law)	[506] (pp. 2, 127), [505] (p. 204), [206] (p. 38)	[222] (p. 58), [211] (p. 4)
